# Pleiotropic functions of catabolite control protein CcpA in Butanol-producing *Clostridium acetobutylicum*

**DOI:** 10.1186/1471-2164-13-349

**Published:** 2012-07-30

**Authors:** Cong Ren, Yang Gu, Yan Wu, Weiwen Zhang, Chen Yang, Sheng Yang, Weihong Jiang

**Affiliations:** 1 Key Laboratory of Synthetic Biology, Institute of Plant Physiology and Ecology, Shanghai Institutes for Biological Sciences, Chinese Academy of Sciences, 300 Fenglin Road, Shanghai 200032, China; 2 School of Chemical Engineering and Technology, Tianjin University, Tianjin, 300072, China; 3 Shanghai Research and Development Center of Industrial Biotechnology, Shanghai, 201201, China

**Keywords:** CcpA, Pleiotropic regulator, Acidogenesis and solventogenesis, Sporulation

## Abstract

**Background:**

*Clostridium acetobutylicum* has been used to produce butanol in industry. Catabolite control protein A (CcpA), known to mediate carbon catabolite repression (CCR) in low GC gram-positive bacteria, has been identified and characterized in *C. acetobutylicum* by our previous work (Ren, C. et al. 2010, Metab Eng **12:**446**–**54). To further dissect its regulatory function in *C. acetobutylicum*, CcpA was investigated using DNA microarray followed by phenotypic, genetic and biochemical validation.

**Results:**

CcpA controls not only genes in carbon metabolism, but also those genes in solvent production and sporulation of the life cycle in *C. acetobutylicum*: *i*) CcpA directly repressed transcription of genes related to transport and metabolism of non-preferred carbon sources such as d-xylose and l-arabinose, and activated expression of genes responsible for d-glucose PTS system; *ii*) CcpA is involved in positive regulation of the key solventogenic operon *sol* (*adhE1-ctfA-ctfB*) and negative regulation of acidogenic gene *bukII*; and *iii*) transcriptional alterations were observed for several sporulation-related genes upon *ccpA* inactivation, which may account for the lower sporulation efficiency in the mutant, suggesting CcpA may be necessary for efficient sporulation of *C. acetobutylicum*, an important trait adversely affecting the solvent productivity.

**Conclusions:**

This study provided insights to the pleiotropic functions that CcpA displayed in butanol-producing *C. acetobutylicum*. The information could be valuable for further dissecting its pleiotropic regulatory mechanism in *C. acetobutylicum*, and for genetic modification in order to obtain more effective butanol-producing *Clostridium* strains.

## Background

CcpA is a conserved regulator protein in gram-positive bacteria, which was first characterized as a transcriptional regulator responsible for glucose repression on α-amylase synthesis in *Bacillus subtilis*, a model firmicute bacterium [1,2]. The later studies have demonstrated that *B. subtilis* CcpA is a master repressor or activator to many cellular processes, such as sugar transport [3,4], glycolysis [5,6], ammonium assimilation [7,8] and d-xylose and l-arabinose metabolism [9,10]. In *Clostridium* species, CcpA was reported to be involved in various cellular processes, such as glucose repression on d-xylose metabolism in *C. acetobutylicum* by our laboratory [11]; also efficient sporulation and enterotoxin gene regulation [12], biofilm formation [13] and gliding in pathogen *C. perfringens*[14]; and repression of toxin gene expression in *C. difficile*[15].

Gram-positive *C. acetobutylicum* has been used to produce organic solvents through a so-called ABE (acetone-butanol-ethanol) fermentation process [16,17]. Among several solvents that ABE fermentation process produces, butanol has attracted significant attention in recent years as a potential next-generation liquid fuel [18,19]. Compared to the other existing biofuels, such as ethanol, butanol offers advantages as a gasoline substitute because of its higher energy content and hydrophobicity [20]. *C. acetobutylicum* genome contains one *ccpA* gene [21,22], the product of which shares 42% amino acid identity with that of *B. subtilis*. However, currently very little is known about the regulatory roles of CcpA in *C. acetobutylicum*. Our previous analysis showed that *C. acetobutylicum ccpA* mutant established significant phenotypic changes (e.g., deficiency in acids re-assimilation) compared to its parental strain [11], suggesting that in addition to regulation of d-xylose metabolism, CcpA may be involved in regulation of other cellular processes in *C. acetobutylicum*.

Since CcpA likely exerts pleiotropic regulation in *C. acetobutylicum*, it thus raises an intriguing question, namely whether *C. acetobutylicum* CcpA is involved in regulating specific cellular processes that are closely related to its industry applications, such as acids re-assimilation, solvents forming, sporulation and capability of utilizing non-preferable sugars [19,23-26]. Positive modification of regulation of these cellular processes will result in significant improvement in terms of economic feasibility of industrial-scale butanol production.

In this study, we employed a comparative transcriptome analysis in combination with genetic and biochemical validation to determine the possible regulatory functions of CcpA in *C. acetobutylicum*. The results showed CcpA is an important pleiotropic regulator in *C. acetobutylicum*: *i*) activating genes in d-glucose-specific phosphotransferase system (PTS), enhancing d-glucose utilization; *ii*) mediating carbon catabolite repression on non-preferred carbon sources utilization; *iii*) regulation of acidogenesis and solventogenesis pathways; and *iv*) necessary for efficient sporulation. The study revealed novel aspects of CcpA regulatory functions in *C. acetobutylicum*, offering new targets for further engineering this solventogenic clostridia.

## Results and discussion

### Global changes of transcriptome of *C. acetobutylicum* caused by *ccpA* inactivation

**Figure 1:**
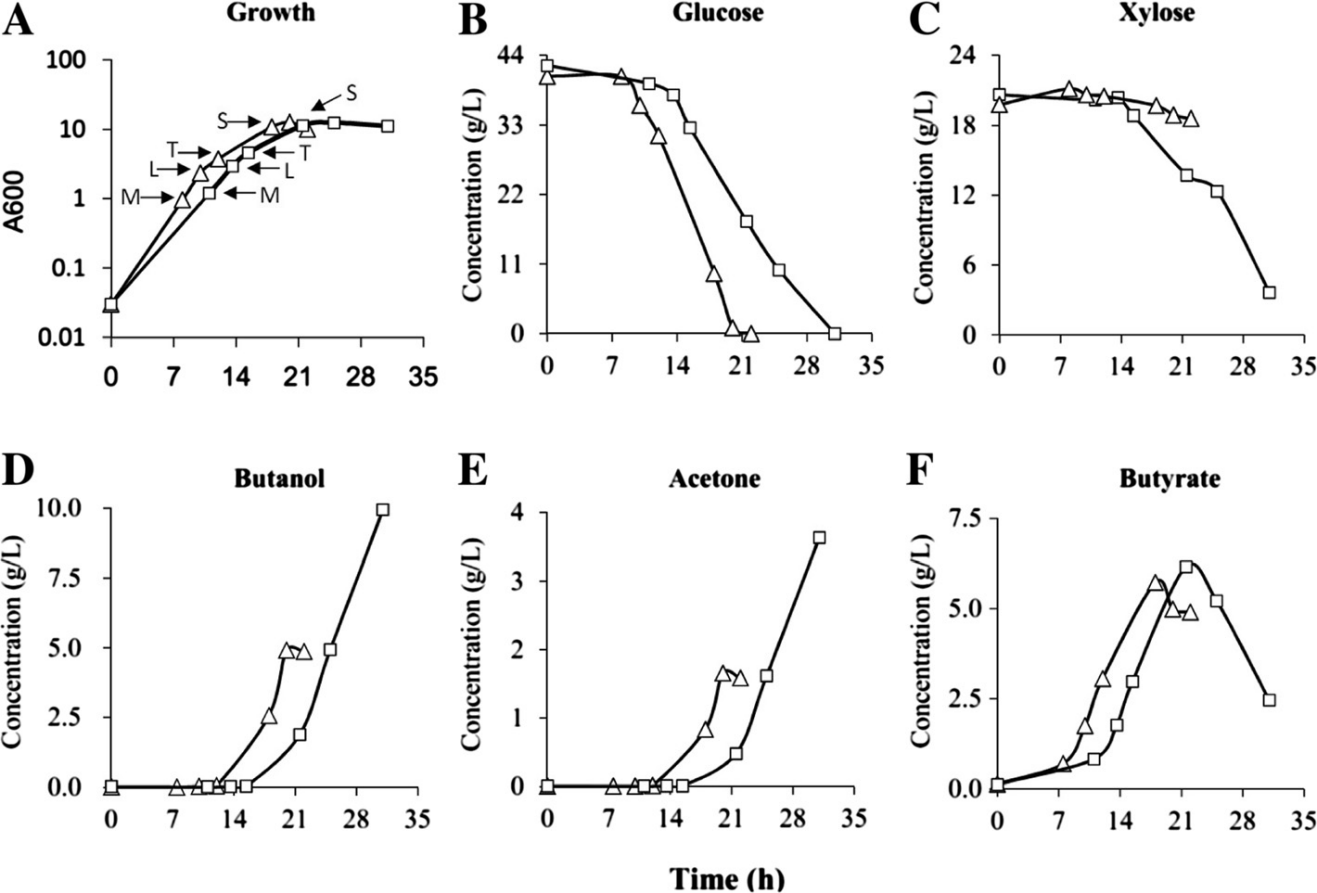
**Comparison of the growth, sugar consumption and production of solvents (butanol and acetone) and butyrate between the 824ccpA (Square) and 824WT (triangle) strains when fermentingd-glucose andd-xylose with pH control. pH value was kept over 5.0 by using 9% (w/v) aqueous ammonia.** Samples (indicated by arrows) for microarray analysis were collected in four time points: middle (M) and late (L)-exponential growth phase; transition phase (T) and stationary phase (S).

Since the regulatory roles of *C. acetobutylicum* CcpA in fermenting d-glucose and d-xylose mixture (simulating lignocellulosic hydrolysates components) are of great interest, these two mono sugars were used as the carbon sources in our fermentation experiments, from which samples were taken for microarray assay. We have previously found that inactivation of the *ccpA* gene caused acids accumulation in *C. acetobutylicum*, resulting in a deficient growth [11]. Therefore, in order to create a normal growth profile for the *ccpA*-inactivated strain (824ccpA), pH was controlled (≥5.0) during the fermentation process for the 824ccpA as well as the wild type strain (824WT) (Figure 1). Microarray analysis was performed to investigate global control of gene expression by CcpA during growth on d-glucose and d-xylose at four time points, at which *A*_600_ values of the 824WT and 824ccpA were similar (Figure 1A). The time point M (middle exponential phase) and L (late exponential phase) were in acidogenic phase, while the time point T (transition phase) and S (stationary phase) were in shift phase (shifting from acidogenesis to solventogenesis) and solventogenic phase, respectively. In addition, in order to confirm whether the genes involved in carbohydrates uptake and metabolism, which were found to be repressed by CcpA, are also subject to “glucose repression” (also called Carbon Catabolite Repression, CCR), the transcriptional levels of these genes in 824WT were also investigated using microarray in the presence (time point S) and absence (time point S2 and S3) of d-glucose (Additional file 1A: Figure S1).

#### (a) Overview of DNA microarray analysis

Comparative transcriptomic analysis of the 824ccpA and 824WT showed that 636, 596, 608 and 936 genes were differentially expressed (change fold ≥2.0) in the phase M, L, T and S, respectively (Additional file 2: Table S1). In addition, more up-regulated genes than down-regulated genes were found throughout the phase M, L and T, while nearly equal numbers of up- and down-regulated genes identified in phase S (Table 1).

**Table 1:** **Numbers of genes (with known or predicted functions) that exhibited significant up- or down-regulation after inactivation of *ccpA***

**Function category**	**No. of genes affected by *ccpA *inactivation**							
	**M (Middle exponential phase)**		**L (Late exponential phase)**		**T (Transition phase)**		**S (Stationary phase)**	
	**Up-regulation**	**Down-regulation**	**Up-regulation**	**Down-regulation**	**Up-regulation**	**Down-regulation**	**Up-regulation**	**Down-regulation**
G. Carbohydrate transport and metabolism	96	9	96	10	84	10	103	9
E. Amino acid transport and metabolism	38	28	9	38	31	52	42	18
H. Coenzyme transport and metabolism	21	12	10	11	16	9	22	22
R. General function prediction only	20	19	20	24	24	23	24	36
K. Transcription	19	13	20	13	22	13	25	22
F. Nucleotide transport and metabolism	14	4	14	1	13	3	11	7
T. Signal transduction mechanisms	11	15	12	7	11	19	19	10
C. Energy production and conversion	10	22	5	15	9	11	16	12
I. Lipid transport and metabolism	9	3	9	3	9	2	11	14
O. Posttranslational modification, protein turnover, chaperones	9	10	2	17	5	16	7	8
N. Cell mobility	8	12	11	4	7	12	14	3
M. Cell wall/membrane biogenesis	8	16	10	10	9	20	13	35
P. Inorganic ion transport and metabolism	8	22	5	25	11	17	25	18
J. Translation	6	5	10	3	5	3	6	38
L. Replication, recombination and repair	5	2	9	1	3	0	4	12
Q. Secondary metabolites biosynthesis, transport and catabolism	4	1	3	1	3	1	3	6
V. Defense mechanisms	2	3	2	1	4	2	5	7
D. Cell cycle control, mitosis and meiosis	1	1	2	1	1	1	2	4
U. Intracellular trafficking and secretion	1	0	1	0	2	0	4	4
S. Function unknown	11	22	12	21	13	20	18	37
Not in COGs	93	57	115	46	75	50	124	149
Total	370	266	352	244	330	278	465	471

The differentially expressed genes could be grouped into 19 subsets with known or predicted functions (Table 1). The majority of these genes are related to core metabolism (subset C, E, F, G, and H) (Table 1). The genes (in category G) responsible for carbohydrate transport and metabolism account for a large percentage of total genes with differential expression, occupying 16.5%, 17.8%, 15.5% and 12.0% in phase M, L, T and S, respectively (Table 1).

#### (b) qRT-PCR validation for microarray quality

A subset of 15 genes was selected for qRT-PCR validation. Among these genes, 9 (*xylT, xylB, tkt1, abrB1941, bukII,* CAC3319*, lacR, araD and ptk*) were found to be up-regulated according to the microarray data, 5 (*abrB3647,* CAC1653, *ctfB, glcG*, and *abrB310*) were down-regulated, and the gene *cysC* was down-regulated in the middle exponential phase and up-regulated in the transition phase instead. An obvious positive correlation can be detected between qRT-PCR and microarray results for these genes (Table 2), suggesting a good quality of microarray data.

**Table 2:** **Correlation between microarray and qRT-PCR**^**†**^**results**

**Gene locus**	**Gene name**	**Function of gene product**	**Change fold (824ccpA/824WT)**			
			**Middle exponential phase (M)**		**Stationary phase (S)**	
			**Microarray**	qRT-PCR*	**Microarray**	qRT-PCR*
CAC1345	*xylT*	l-arabinose-proton symporter	71.6	92.52 ± 2.55	147.39	326.54 ± 176.86
CAC2612	*xylB*	Xylulose kinase	26.9	20.4 ± 4.00	17.9	21.21 ± 0.02
CAC1348	*tkt1*	Transketolase	5.6	4.42 ± 0.23	7.9	6.4 ± 0.21
CAC1941	*abrB1941*	Stationary/sporulation gene regulator	5.1	89.17 ± 7.31	1.62	1.62 ± 0.30
CAC1660	*bukII*	Butyrate kinase II	4.3	7.03 ± 2.02	5.2	10.79 ± 0.04
CAC3319	*-*	Signal transduction histidine kinase	3.95	2.7 ± 1.01	3.43	2.41 ± 0.30
CAC2966	*lacR*	Lactose phosphotransferase system repressor	1.95	6.88 ± 1.43	1.13	15.49 ± 5.61
CAC3647	*abrB3647*	Stationary/sporulation gene regulator	0.67	0.47 ± 0.05	0.48	0.61 ± 0.11
CAC1653	*-*	Cell wall biosynthesis glycosyltransferase	0.43	0.34 ± 0.10	0.56	0.41 ± 0.11
CAP0164	*ctfB*	Butyrate-acetoacetate COA-transferase subunit B	0.27	0.17 ± 0.01	1.00^§^	0.24 ± 0.06
CAC0103	*cysC*	Adenylylsulfate kinase	0.25	0.04 ± 0.01	182	49.14 ± 41.18
CAC0570	*glcG*	PTS enzyme II, ABC component	0.16	0.05 ± 0.02	0.19	0.34 ± 0.14
CAC0310	*abrB310*	Stationary/sporulation gene regulator	0.14	0.62 ± 0.08	0.18	0.59 ± 0.22
CAC1341	*araD*	l-ribulose-5-phosphate 4-epimerase	2.29	2.11 ± 0.50	5.81	2.33 ± 1.80
CAC1343	*ptk*	Phosphoketolase	1.12	1.84 ± 0.59	2.87	2.35 ± 0.71

^†^The wild-type (824WT) and *ccpA*-inactivated mutant strain (824ccpA) were cultured in P2 medium using 40 g/L d-glucose and 20 g/L d-xylose as the carbon sources. Cells were harvested at middle exponential phase (A_600_ ≈ 1.0) and early stationary phase (A_600_ ≈ 12.0).

^*^ The data were mean ± standard deviation (SD) of two independent biological replicates.

^§^ The microarray result of the *ctfB* gene at S phase was inaccurate because its expression level exceeded the detection upper limit.

### Prediction of CcpA binding sites based on microarray data

It has been known that CcpA can directly regulate its target genes by binding to the CRE sites [27-29]. Here, such a CRE motif (WTGWAAACGWTWWCAW) was retrieved for the up-regulated genes after *ccpA* inactivation in *C. acetobutylicum* by using Gibbs Motif Sampler program [30]. The putative CRE motif of *C. acetobutylicum* showed high similarity (*p*-value =4.09 × 10^-8^) to the previously identified *B. subtilis* CRE motif according to the motif comparison utility TOMTOM [3], indicating that these CRE sequences are reliable. In the contrary, despite that some down-regulated genes were found to harbor putative CRE sites (Addition file 3), no such a conserved motif can be retrieved from the promoter sequences of these genes, thus indicating that the CcpA-binding sites of most of the down-regulated genes are atypical or they might be indirectly regulated by CcpA.

**Figure 2:**
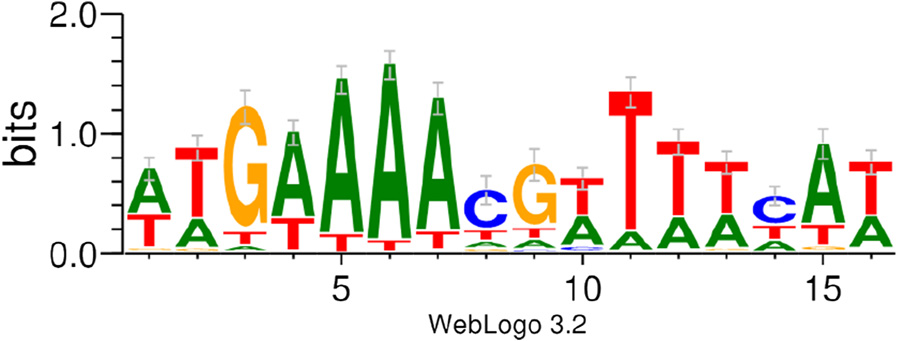
**Visualization of *C. acetobutylicum *CRE site consensus.** The consensus sequence was generated from all of the predicted CRE sites.

Next, we adopted HMM (Hidden Markov Model) [31], which was built based on the above CRE sequences, to search all potential CcpA-binding CRE sites in *C. acetobutylicum*. The bit score assigned by HmmSearch program reflects the similarity of a sequence match to a profile Hidden Markov Model [32]. The result (Additional file 3: Table S2) showed that 148 and 224 putative CRE sites were detected upstream of the Open Reading Frame (ORF) and within ORF region, respectively. The consensus motif of these CRE sites was visualized in Figure 2.

### Effect of CcpA on carbohydrates uptake and metabolism

Large number of genes, involved in carbohydrate uptake and catabolism, were affected by *ccpA* inactivation in *C. acetobutylicum* (Table 1). A total of 119 genes exhibited significant (change fold ≥2.0) alterations in expression levels, of which 108 were up-regulated and 11 were down-regulated (Additional file 4: Table S3 and Additional file 5: Table S4). Expressional levels of most of the CcpA-repressed genes (98 genes) in the 824WT were up-regulated when d-glucose was depleted from the glucose-xylose mixture (Additional file 1: Figure S1), indicating that CcpA did mediate carbon catabolite repression (CCR) on these 98 genes. The majority (70%) of the CcpA-repressed genes or operons (namely those showed up-regulation after *ccpA* inactivation by microarray analysis) harbored CRE sites in their upstream or ORF regions (Additional file 4: Table S3), which is consistent with the previous observations in *Bacillus* and *Lactobacillus species*[28,33,34].

#### (a) PTS sugar transport and metabolism

**Figure 3:**
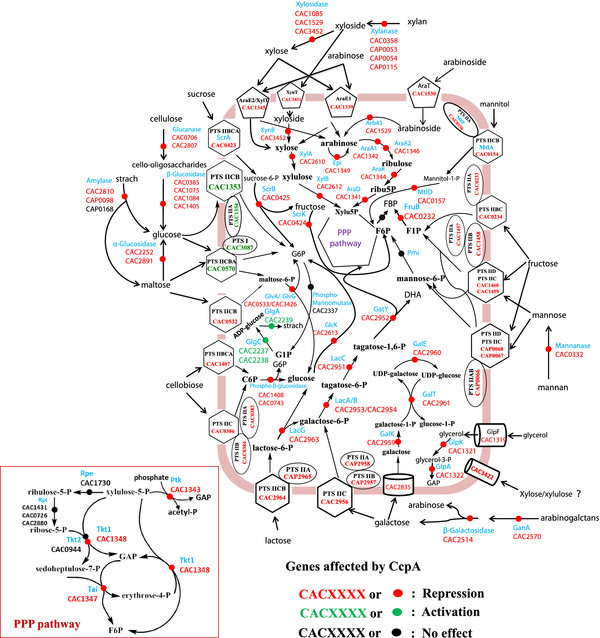
**Schematic of gene targets, related to carbohydrates utilization or synthesis pathways, regulated by CcpA based on the microarray analysis.** Abbreviations are as follows: XynT, sugar/Na^+^(H^+^) simporter; AraE1/AraE2/XylT, sugar-proton symporter; XylA, xylose isomerase [43]; XynB, Beta-xylosidase; XylB, xylulose kinase; Tkt1/Tkt2, transketolase; Tal, transaldolase; AraA1/AraA2, L-arabinose isomerase; AraK, ribulokinase [44]; AraD, L-Ribulose-5-phosphate 4-epimerase; ScrA, beta-glucosides specific PTS IIBCA component; ScrB, sucrase-6-phosphate hydrolase; ScrK, fructokinase; MltF, mannitol-specific PTS IIA component; MtlA, mannitol-specific PTS IIBC component; MtlD, mannitol-1-phosphate 5-dehydrogenase; FruB, 1-phosphofructokinase; Pmi, phosphomannose isomerase; GlvA, maltose-6-phosphate glucosidase; GlgC, glucose-1-phosphate adenylyltransferase; GlgA, starch synthase; LacG, 6-phospho-beta-galactosidase; LacA, galactose-6-phosphate isomerase subunit A; LacB, galactose-6-phosphate isomerase subunit B; LacC, tagatose-6-phosphate kinase; GatY, tagatose-bisphosphate aldolase; GalK, galactokinase; GalT, galactose-1-phosphate uridylyltransferase; GalE, UDP-galactose 4-epimerase; GlpK, glycerol kinase; GlpA, glycerol-3-phosphate dehydrogenase; GanA, arabinogalactan endo-1,4-beta-galactosidase; GlpF, glycerol uptake facilitator protein; GlcK, glucokinase; GlvG, 6-phospho-alpha-glucosidase; GlgC, glucose 1-phosphate adenylyltransferase; GlgA, glycogen synthase; AraT , arabinosides-proton symporter [44]; Arb43, Alpha-L-arabinofuranosidase II precursor [44]; Ptk, phosphoketolase; Rpi, ribose 5-phosphate isomerase; Rpe, aldose-1-epimerase; Epi, arabinose mutarotase; GAP, glyceraldehyde 3-phosphate; F1P, fructose 1-phosphate; F6P, fructose 6-phosphate; FBP, fructose 1,6-biphosphate; G6P, glucose 6-phosphate; G1P, glucose 1-phosphate; C6P, cellobiose 6-phosphate; Xylu5P, xylulose 5-phosphate; DHA, dihydroxyacetone.

Microarray results showed that many genes responsible for typical PTS transporters and enzymes catalyzing metabolism of non-glucose carbohydrates, such as fructose, mannose, cellobiose, galactose, lactose [35], sucrose [36], maltose and mannitol, exhibited higher expression in the 824ccpA compared to the 824WT (Additional file 4 and Figure 3). Since most of these genes or operons were predicted to harbor CcpA-binding sites (CRE sites) in the promoters or ORF regions (Additional file 4), it seems that CcpA can directly repress them through binding to these CRE sites. Among these genes, CAC3425 and CAC3427 may deserve some attention. Although these two genes have been annotated as glucose-specific PTS components in the previous studies [37-39], they might be involved in transporting other PTS sugars because: *i*) these two genes were subject to CcpA-mediated glucose repression (Additional file 1), indicating that they unlikely act as glucose-specific PTS components; *ii*) according to the genome context analysis [40,41], they are included in one operon in combination with *pagL* (CAC3426, encoding 6-phospho-alpha-glucosidase), which could not be induced by d-glucose [42].

In addition, a CRE site was found in the ORF region of CAC1454 (Additional file 4), which is a histidine kinase-encoding gene embraced in a transcription unit CAC1453-CAC1454-CAC1455-CAC1456. Within this unit: gene CAC1453, which was up-regulated after *ccpA* inactivation (Additional file 4), is annotated as a periplasmic ribose-binding component of ABC transporter in the GenBank sequence database provided by the National Center for Biotechnology Information (NCBI), whereas annotated as a periplasmic fructose-binding protein in database SEED ( http://theseed.uchicago.edu/FIG/index.cgi) [45]; gene CAC1455, encoding a periplasmic fructose-binding protein component of signal transduction system LevQ (from SEED database), was also found to be related to fructose utilization [46]. Therefore, this gene cluster is likely responsible for fructose metabolism.

**Figure 4:**
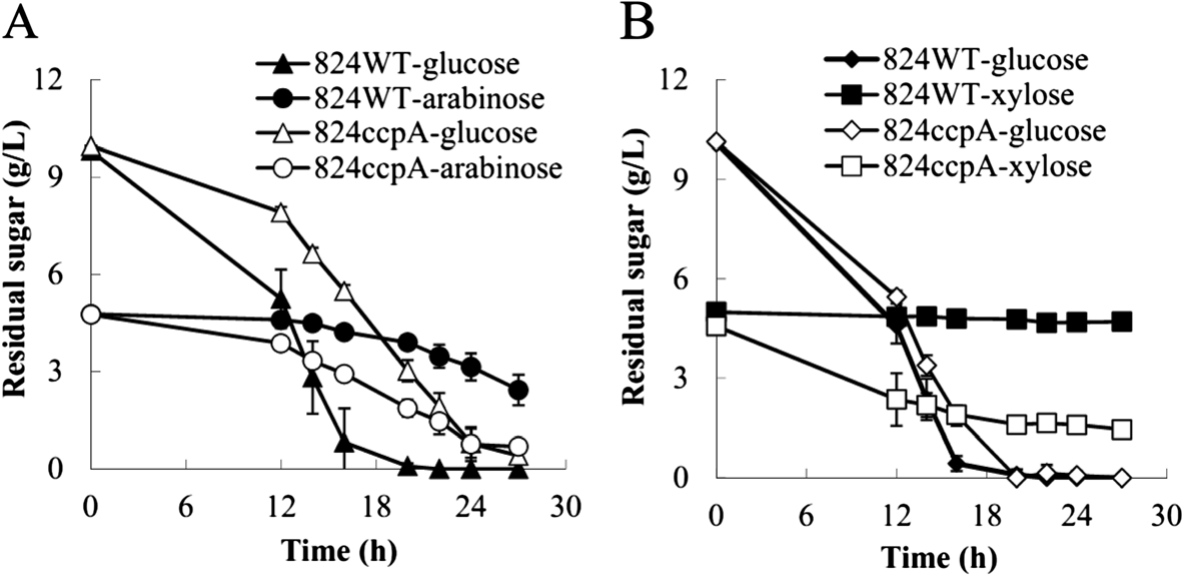
**Comparison of sugar consumption between the 824WT and 824ccpA strain in fermenting mixed sugars (A,d-glucose andl-arabinose; B,d-glucose andd-xylose).**

In contrast, gene *glcG* (CAC0570), CAC1353 and CAC1354, encoding d-glucose PTS enzyme II components, were all significantly down-regulated after *ccpA* inactivation (Additional file 5: Table S4 and Figure 3). This observation suggested that CcpA may be necessary for the efficient expression of d-glucose PTS genes in *C. acetobutylicum*. It should be noted that the importance of gene *glcG* in d-glucose uptake has been previously described [47]. Our previous study also showed that its inactivation has potential value of alleviating glucose repression on non-preferable sugars metabolism [48]. Besides, the gene *ptsI* (CAC3087), encoding phosphoenolpyruvate-protein kinase (PTS enzyme I), was also significantly down-regulated here (Additional file 5 and Figure 3). Therefore, the down-regulation of *glcG*, CAC1353-1354 and *ptsI* might be responsible for the slower d-glucose consumption rate of the 824ccpA compared to the 824WT (Figure 1B and Figure 4), thus resulting in alleviation of glucose repression on non-preferable sugars [48,49]. In addition, a CRE site was found in the intergenic region of CAC1353 and CAC1354, two divergently transcribed genes, whereas no such sites were found for gene *glcG* and *ptsI* (Additional file 5). The results indicated that CcpA might directly regulate either CAC1353 or CAC1354 or both, but affect the expression of *glcG* and *ptsI* indirectly or through binding to atypical CRE sites.

The *cggR* operon-like gene cluster CAC0708-*gapCpgktpiApgmeno* (CAC0708-0713), which is highly similar to the *B. subtilis cggR* operon (*cggRgapApgktpiApgm*) and encodes several key enzymes for glycolysis [6,50], did not exhibit obvious difference in the transcriptional change between the 824ccpA and 824WT (Additional file 2). This result is quite different to what has been found in *B. subtilis*, in which all these genes were activated by CcpA. In addition, gene CAC2018, encoding an aldehyde: ferredoxin oxidoreductase for catalyzing the conversion from glyceraldehyde-3-phosphate to glycerate-3-phosphate, was the only glycolysis-related gene which exhibited significant up-regulation after *ccpA* inactivation (Additional file 4), however, no typical CRE site could be found in either the upstream region or the ORF of CAC2018.

#### (b) Polysaccharides metabolism

The expression of the putative granulose-synthetic gene cluster CAC2237-CAC2240 (CAC2237: *glgC*, encoding glucose-1-phosphate adenylyltransferase; CAC2238: *glgC*, encoding ADP-glucose pyrophosphorylase; CAC2239: *glgA*, encoding glycogen synthase; CAC2240, encoding cyclomaltodextrin glucanotransferase domain-containing protein) [51] was significantly down-regulated by *ccpA* inactivation (Additional file 5 and Figure 3), however, no putative CRE sites can be found in its upstream region. On the contrary, several genes responsible for starch degradation exhibited significant up-regulation after *ccpA* inactivation: an α-amylase gene *amyA1* (CAP0098), two α-glucosidase genes (CAC2252, CAC2891) and a possible glucoamylase gene (CAC2810) (Additional file 4 and Figure 3). These four genes all have putative CRE sites (Additional file 4) in their upstream regions or ORFs, suggesting that CcpA might regulate them directly. As to another starch-degrading gene *amyA2* (CAP0168), no obvious changes were found in the expression level at the first three phases, and only a 2.8-fold increase occurred at S phase (Additional file 2), which was much lower than fold-changes of other starch-degrading genes. Considering that no potential CRE site can be found upstream of the *amyA2* gene, and the fact that gene *amyA2* showed no obvious change in transcriptional level when cells were cultivated with and without starch [37] and *amyA2*-encoded α-amylase could be readily purified from the cells grown on d-glucose [52], this gene is unlikely subject to CcpA-dependent glucose repression in *C. acetobutylicum*. It should be noted here that *amyA1* and *amyA2* likely act as the major genes responsible for starch degradation in *C. acetobutylicum*, because the loss of the megaplasmid pSOL1 (harboring gene *amyA1* and *amyA2*) caused serious defect in starch degradation [53].

It is known that cellulases and several scaffolding proteins organize as cellulosome in some *Clostridium* species [54]. *C. acetobutylicum* also harbors a series of cellulosome-encoding genes, but its cellulosome did not exhibit activity required to degrade crystalline cellulose [55]. We found that two glucanase-encoding genes, namely CAC0706 (encoding endo-1, 4-beta glucanase) and CAC2807 (encoding endo-1, 3(4)-beta-glucanase), and several β-glucosidase-encoding genes (CAC0385, CAC1075, CAC1084 and CAC1405) were all up-regulated in the 824ccpA in comparison to the 824WT (Additional file 4 and Figure 3). Putative CRE sites were also identified in the upstream or ORF regions of four out of these six genes, i.e. CAC0706, CAC1075, CAC1084 and CAC1405 (Additional file 4). Moreover, we noticed that these four genes all showed up-regulation in the 824WT strain when d-glucose was depleted (Additional file 1), suggesting that they were subject to CcpA-mediated glucose repression. Many other genes responsible for cellulose degradation (CAC0910–CAC0919, encoding cellulosome scaffolding proteins; CAP0010, encoding beta-glucosidase; seventeen genes encoding endoglucanase, namely CAC0214, CAC0215, CAC0216, CAC0537, CAC0690, CAC0825, CAC0826, CAC0911, CAC0912, CAC0913, CAC0915, CAC0916, CAC0917, CAC0918, CAC1563, CAC2556, CAC3469) showed no differential expression after *ccpA* inactivation, indicating that they may be not subject to CcpA-mediated regulation.

*C. acetobutylicum* is capable of degrading xylan under some specified conditions [56], and a total of fourteen genes were found to be related to hemicellulose degradation and metabolism [21]. We observed that inactivation of *ccpA* resulted in up-regulation in transcriptional levels of genes responsible for xylan degradation, including CAP0053-CAP0054 operon (xylanase and xylanase/chitin deacetylase family protein), CAP0114-CAP0115 (glycosyl hydrolase and endo-1,4-beta-xylanase), CAC0358 (xylanase/chitin deacetylase), CAC1085 (alpha-xylosidase), *arb43* (CAC1529, encoding alpha-L-arabinofuranosidase II precursor) and *araT* (CAC1530, encoding sugar-proton symporter, possibly for arabinosides) (Additional file 4 and Figure 3). In addition, two genes (CAC2570, CAC2514) and gene CAC0332, encoding putative arabinogalactan-degrading enzymes and mannanase, respectively, all exhibited up-regulation after *ccpA* inactivation (Additional file 4). Possible CRE sites were identified upstream of all these genes or operons except for gene CAC0358 (Additional file 4). Therefore, in combination with the observation that expression of these genes was repressed in the presence of d-glucose (Additional file 1), the results here strongly suggested that they are subject to CcpA-dependent CCR.

#### (c) Pentose transport and metabolism

According to the previous studies [43,44,57], we illustrated the genomic organization of genes for pentose utilization in *C. acetobutylicum* ATCC 824 (Additional file 6:Figure S2). A large number of genes involved in uptake and metabolism of pentose, such as d-xylose and l-arabinose, showed differential expression after inactivation of the *ccpA*. Two gene clusters, namely CAC2610-CAC2612 and CAC1339-CAC1349, are responsible for d-xylose and l-arabinose uptake and metabolism in *C. acetobutylicum*[21].

Gene *xylA* (CAC2610) and gene *xylB* (CAC2612) have been experimentally confirmed as the primary candidates encoding d-xylose isomerase and xylulokinase [43], respectively. These two genes, found to be co-expressed as one operon with CAC2611 (unpublished data), were significantly up-regulated after *ccpA* inactivation (Additional file 4 and Figure 3) and were subject to glucose repression (Additional file 1). In addition, two putative CRE sites were found in this operon: one is in the upstream region of *xylB* and the other is in the ORF of *xylA* (Additional file 4 and Additional file 6). When fermentation was performed using mixed d-glucose and d-xylose as the carbon sources, improved d-xylose utilization was observed by the 824ccpA compared to the 824WT (Figure 4B).

The CAC1339-CAC1349 cluster contains most of the genes necessary for l-arabinose uptake and metabolism, including *araE1/araE2* (CAC1339 and CAC1345, encoding l-arabinose-proton symporter), *araA1/araA2* (CAC1342 and CAC1346, encoding l-arabinose isomerase), *araK* (CAC1344, encoding ribulokinase) [44], *araD* (CAC1341, encoding l-ribulose-5-phosphate 4-epimerase), *epi* (CAC1349, encoding aldose 1-epimerase) and three genes associated with Pentose Phosphate Pathway (PPP), namely *ptk* (CAC1343, phosphoketolase), *tal* (CAC1347, encoding transaldolase) and *tkt1* (CAC1348, encoding transketolase) [37]. Microarray results showed that, except for CAC1340-CAC1343, all genes in this locus (*araE1*, *araK*, *araE2*, *araA2*, *tal, tkt1* and *epi*) exhibited much higher transcriptional level in the 824ccpA versus the 824WT at all four phases (Additional file 4 and Figure 3). Obviously, these genes (*araE1*, *araK*, *araE2*, *araA2*, *tal*, *tkt1* and *epi*) are subject to glucose repression (Additional file 1). One putative CRE site was found inside the ORF of *araK* and upstream of *araE2*, respectively, while there were two CRE sites located in the upstream region of *araE1* (Addition file 4 and Addition file 6). Gene CAC1340, encoding a transcriptional repressor AraR of l-arabinose operon [57], showed no change in transcriptional level between the 824ccpA and 824WT. Gene set *araD-araA1-ptk* (CAC1341-1343) displayed only a slightly differential expression (Table 2 and Additional file 4), although a putative CRE was located in the intergenic region of gene *araR* and gene *araD* (Additional file 4 and Additional file 6). We noticed that the *araD-araA1-ptk* was weakly induced by d-xylose whereas strongly induced by l-arabinose [37], which indicated that the slight transcriptional change of *araD-araA1-ptk* observed here might be attributed to the absence of a strong inducer, namely l-arabinose. We thereby investigated the expression of *araD-araA1-ptk* in both 824WT and 824ccpA in the presence of d-glucose and l-arabinose. The result showed that the transcriptional levels of both *araD* and *ptk* were much higher (>60 fold) in the 824ccpA compared to those of the 824WT (Additional file 7: Figure S3), demonstrating that this gene set (*araD-araA1-ptk*) was subject to CcpA-dependent glucose repression and l-arabinose induction. A recent study has identified three AraR-binding sites in the upstream region of *araD-araA1*[44], indicating a strong repression of this gene set by AraR according to the AraR’s action mode in *B. subtilis*[58]. Moreover, AraR exhibited higher affinity for the upstream region of gene *ptk* than that of operon *araK-araE2-araA2-tal-tkt1-epi*[44]. Therefore, the reason for the insufficient induction of d-xylose to the *araD-araA1-ptk* here could be that d-xylose is not enough to release such a repression of AraR on the gene set *araD-araA1-ptk*. In addition, only slight transcriptional change of gene *araR* was found in the 824ccpA compared to the 824WT when fermenting l-arabinose and d-glucose mixture (Additional file 7). In combination with the observation that *araR* was not subjected to glucose repression (Additional file 7), we thus speculated that *araR* is not subject to CcpA-dependent CCR. Considering that the genes *araE1*, *araD-araA1-ptk* and *araK-araE2-araA2-tal-tkt1-epi* are related to l-arabinose metabolism [37], their up-regulation was expected to enable an improved l-arabinose utilization in the presence of d-glucose. To confirm this speculation, we investigated the 824ccpA and the 824WT in fermenting the mixture of d-glucose and l-arabinose. As shown in Figure 4A, compared to the 824WT, the 824ccpA exhibited a significantly enhanced l-arabinose consumption.

**Figure 5:**
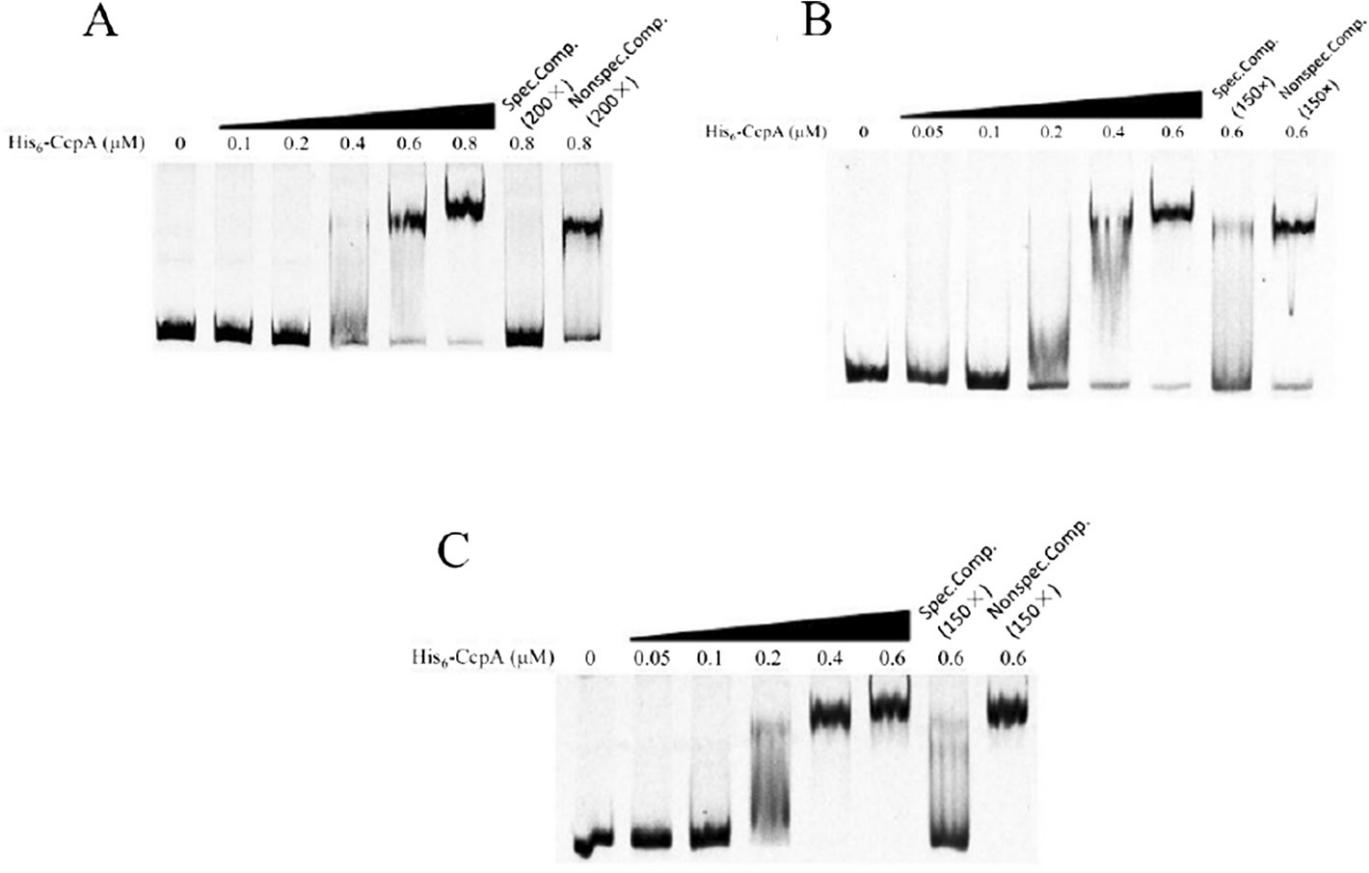
**EMSA to assess the interaction of *C. acetobutylicum *CcpA with (A) *xylB *promoter region P*xylB*, (B) *araE1 *promoter region P*araE1 *and (C) upstream region (non-coding region, P*sol*) of the *sol *operon.** His_6_-CcpA with different concentrations (0–0.8 μM, 0–0.6 μM and 0–0.6 μM for P*xylB*, P*araE1* and P*sol*, respectively) and 2 nM of Cy5-labelled PCR fragments were used. The PCR fragment of gene CAC1790 was used as the non-specific competitor. Abbreviations: Spec. comp., specific competitor; Nonspec. comp., non-specific competitor.

Putative CRE sites were identified in the promoter or ORF regions of these d-xylose and l-arabinose pathway genes, including *araE1*, *xylB*-CAC2611-*xylA*, *araD-araA1-ptk* and *araK-araE2-araA2-tal-tkt1-epi* (Additional file 4 and Additional file 6). To further determine whether CcpA can directly regulate these genes, we performed electrophoretic mobility shift assay (EMSA) analysis. The DNA fragments of *xylB* and *araE1* promoter regions were chosen as the candidates. A substantial DNA band shift was observed in both cases and typical protein concentration dependent binding trends were also observed (Figure 5A and 5B). The band shift was suppressed with un-labelled DNA competitor but not in the presence of same amount of non-specific competitor (gene CAC1790 fragment) (Figure 5A and 5B). Thus, the binding of CcpA to the promoter regions of *xylB* and *araE1* will repress their transcription, resulting in inhibition of d-xylose and l-arabinose consumption when d-glucose was present (Figure 4). These results demonstrated that CcpA directly repressed the expression of genes *xylB*-CAC2611-*xylA* and *araE1*, which are key genes for d-xylose and l-arabinose utilization, respectively.

### Impact of CcpA in acidogenesis and solventogenesis

*C*. *acetobutylicum* possesses two distinct phases during the typical ABE fermentation process: acidogenic phase when acetic acid and butyric acid are produced and secreted, and solventogenic phase when the acids are re-assimilated for the production of three chemicals (acetone, butanol and ethanol) [59,60]. Due to the importance of these two physiological processes in ABE production, increasing attentions have been paid to identify related structural genes (e.g., *sol* operon and *adc* gene) as well as some transcription factors (e.g., Spo0A, AbrB) [59-64]. In spite of these progresses, the regulatory mechanism underlying the acids secretion and solvents formation as well as their metabolic shift are still not well understood. Our previous study in the 824ccpA strain revealed that *ccpA* inactivation issued a great impact on the solventogenesis [11]. Microarray analysis in this study showed that CcpA, besides mediating carbon catabolite repression in gram-positive bacteria, might be involved in regulation of acids and solvents formation as well as their shift in *C. acetobutylicum*. The *sol* operon (*adhE1-ctfA-ctfB*), responsible for acids re-assimilation and alcohol production [65], gene *abrB310*, an essential regulator for transition between acidogenic and solventogenic phase [63], and gene *bukII*, a second butyrate kinase related to butyrate formation [66,67], were all found to be regulated by CcpA. Inactivation of *ccpA* gene resulted in obvious changes in acids and solvents forming as well as the transition of these two stages [11].

#### (a) CcpA inactivation caused down-regulation of the *sol* operon

**Figure 6:**
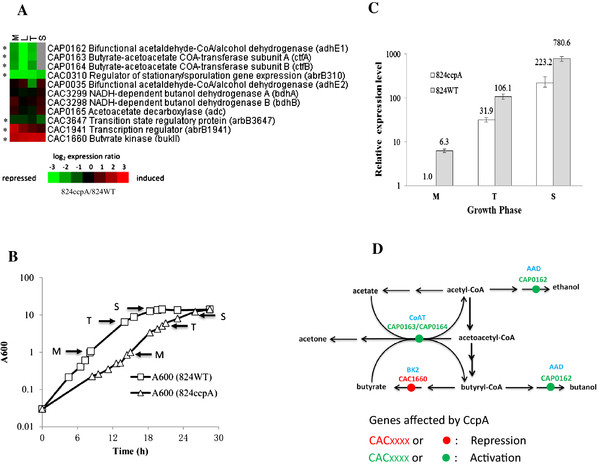
**Role of CcpA in acidogenesis and solventogenesis.** (**A**) Transcriptional profile of genes involved in acidogenesis and solventogenesis after *ccpA* inactivation. Red and green color represented the genes exhibiting increased and decreased expression level after *ccpA* inactivation, respectively. The genes showed obvious transcriptional changes (change-fold ≥ 2.0) were indicated in asterisks besides the heatmap. The data of *sol* operon (*adhE1*-*ctfA*-*ctfB*) at S time point was not included here because its expressional level exceeded the upper limit of microarray’s detection range. (**B**) Growth curve for qRT-PCR validation. (**C**) qRT-PCR validation for the expressional change of *sol* operon after *ccpA* inactivation. Cells were cultured in P2 medium containing 40 g/L d-glucose and 20 g/L d-xylose as the carbon sources. pH was controlled over 5.0 during the whole fermentation period by using aqueous ammonia. Cells were harvested at middle exponential phase M (A_600_ ≈ 1.0), transition phase T (A_600_ ≈ 8.0) and stationary phase S (A_600_ ≈ 12.0). The *sol* expression level of the 824ccpA strain at phase M was used as the control for normalization. M, middle exponential growth phase; T, acidogenesis-solventogenesis transition phase; S, stationary solventogenesis phase. The reported data were mean ± standard deviation (SD) of three technical replicates. (**D**) Schematic of the genes, for acids synthetic, acids re-assimilation and solvents formation, regulated by CcpA.

The *sol* operon of *C. acetobutylicum* contains three genes, i.e., *adhE1*, *ctfA* and *ctfB*, which are sequentially arranged and transcribed as a tri-cistron under the control of the *adhE1* promoter and the operon has been found to play a key role in alcohol production and acid re-assimilation in *C. acetobutylicum*[65]. Microarray analysis showed that among the genes (*adhE1*, *ctfA*, *ctfB*, *adhE2*, *adc*, *bdhA* and *bdhB*) concerned with branch pathways of solvents formation, only *sol* operon genes showed differential expression after *ccpA* inactivation (Figure 6A). To further confirm expression changes of the *sol* operon, qRT-PCR analysis was performed. The samples were collected at M, T and S time points, representing the acidogenic exponential, acid-to-solvent transition and early solventogenic period (Figure 6B), respectively. As shown in Figure 6C, significantly lower expression of *sol* operon was detected in the 824ccpA versus the 824WT, demonstrating that CcpA is required for normal expression of *sol* operon during both acidogenic and solventogenic phase. Although no typical CRE sites were found in the upstream region of *sol* operon, the EMSA result showed that the CcpA protein was able to interact directly with the *sol* upstream region *in vitro*, and an apparent shift was observed when 0.4 μM CcpA was used (Figure 5C). The CcpA-DNA binding appeared to be specific since no shift was observed in the presence of non-labeled specific competitor, and non-specific competitor (gene CAC1790 fragment) did not affect the CcpA-DNA binding (Figure 5A). Therefore, it seems that there is an atypical CcpA-binding site inside the upstream region of *sol* operon.

It should be noted that, although *ccpA* inactivation lowered the transcriptional level of *sol* operon, the trend that the transcript of *sol* operon gradually increased from M to S phase was similar for the 824WT and 824ccpA strain (Figure 6C), suggesting that some other factors might be involved in regulating *sol* expression. It has been known that the phosphorylated Spo0A, a transcription factor, could activate the *sol* expression [61,68-70], and inactivation of the *spo0A* caused a deficient *sol* expression, resulting in a significant decrease in solvent formation [69,71]. Therefore, Spo0A is considered as a “master controller” for solvent production in *C. acetobutylicum*[72]. The previous observations in combination with the results in this study suggested that both CcpA and Spo0A could regulate *sol* operon, which also explained why the *sol* operon was still efficiently induced in the 824ccpA strain (Figure 6C). However, the interaction of CcpA and Spo0A in regulating *sol* operon is still yet to be elucidated.

#### (b) ccpA is involved in glucose repression on *bukII* expression

*C. acetobutylicum* ATCC 824 harbors two butyrate kinase (BK) genes, namely *bukI* (CAC3075) and *bukII* (CAC1660). The *bukI* gene, together with *ptb* gene, forms an operon responsible for producing butyrate, a major organic acid in *C. acetobutylicum*[73]. Although *in vitro* butyrate kinase activity of BKII could be easily detected [66,67], its *in vivo* catalytic activity remained at a much lower level compared to that of BKI [66]. Interestingly, microarray and qRT-PCR results here showed that *ccpA* inactivation caused a significant increase in transcriptional level of the *bukII* (Figure 6A and Table 2) and the expression of *bukII* was repressed by d-glucose in the 824WT (Additional file 8: Figure S4). These observations indicated that *bukII* of *C. acetobutylicum* is a glucose-repressive gene and moreover, such a glucose repression is under CcpA-dependent control. However, no CRE site was found in the upstream region or ORF region of gene *bukII*, and the EMSA result also showed no binding of CcpA to the *bukII* (data not shown). Therefore, it seems that gene *bukII* is not directly regulated by CcpA. In contrast, no change was detected in expression level of the *bukI-ptb* operon after *ccpA* inactivation (Additional file 2). Therefore, the enhanced butyrate formation in the 824ccpA might be partly attributed to the up-regulation of the *bukII*. The up-regulated *bukII* expression in combination with the down-regulation of the *sol* operon (responsible for alcohol production and acids re-assimilation) may result in excess butyrate accumulation as well as a consequent “acid crash” in the 824ccpA, and thereby, the 824ccpA required a pH adjustment during the fermentation process so as to live through the acidogenic period. Once entering into the solventogenic period, the ability of the 824ccpA in acid re-assimilation and solvent production could be restored because of the gradually increased expression of the *sol* operon from then on (Figure 6C). This also resulted in a comparable residual butyrate concentration of the 824ccpA and 824WT at the end of the fermentation (Figure 1).

BK (butyrate kinase)-dependent butyrate formation is an ATP-producing process in *C. acetobutylicum*[73]. Therefore, it is speculated that expression of the *bukII* is only efficiently activated when fermenting some non-preferable sugars (such as d-xylose and l-arabinose) because, compared to d-glucose metabolism, a higher ATP demand is required then [74]. Although it seems that gene *bukII* did not work when fermenting d-glucose, such a CcpA-dependent glucose repression on the *bukII* expression may be a significant regulatory mechanism for *C. acetobutylicum* to respond to different carbohydrate resources.

#### (c) CcpA controls *abrB* expression

Gene *abrB* has been linked to regulation of the shift from exponential to stationary growth phase in *B. subtilis*[75,76]. *C. acetobutylicum* ATCC 824 harbors three *abrB* homologues, namely *abrB310* (CAC0310), *abrB1941* (CAC1941) and *abrB3647* (CAC3647), and these three AbrB protein are highly identical (~70%) to each other. According to microarray result and qRT-PCR validation, three *abrB* genes all exhibited different expression changes after *ccpA* inactivation (Figure 6A and Table 2): the expression levels of the *abrB310* and *abrB3647* were significantly down-regulated during all four phases, while that of the *abrB1941* was only up-regulated in acidogenic phase. The previous study using antisense RNA approach has showed that repression of the *abrB310* expression resulted in a prolonged acidogenic period as well as delayed solvent formation in *C. acetobutylicum*[63]. Therefore, AbrB310 is likely involved in regulation of the shift from acidogenesis to solventogenesis. However, the possible regulatory roles that *abrB3647* and *abrB1941* play as well as the relationship of these three *abrB* genes still need further investigation.

### CcpA protein is required for efficient sporulation in *C. acetobutylicum*

A number of genes related to spore forming exhibited differential expression after *ccpA* inactivation in *C. acetobutylicum*. To further confirm the microarray result, we investigated sporulation formation of the 824ccpA and 824WT strains. After 2 d growth, the 824WT strain produced spores at 2.23% while the 824ccpA did not produce any spores (Table 3). When the growth time prolonged to 8 d, sporulation of the 824WT increased to ~100%, nearly 4.5-fold that of the 824ccpA (22.9%) (Table 3), suggesting that CcpA is required for efficient sporulation in *C. acetobutylicum*.

**Table 3:** Comparison of sporulation phenotype between the 824WT strain and the 824ccpA mutant

**Strain**	**2 d**			**4 d**			**6 d**			**8d**		
	**Total number of CFU**	**Number of spores**	**Sporulation (%)**	**Total number of CFU**	**Number of spores**	**Sporulation (%)**	**Total number of CFU**	**Number of spores**	**Sporulation (%)**	**Total number of CFU**	**Number of spores**	**Sporulation (%)**
824WT	(1.64 ± 1.31) × 10^5^	(3.66 ± 2.59) × 10^3^	2.23	(8.89 ± 0.80) × 10^4^	(5.01 ± 1.29) × 10^4^	56.37	(1.40 ± 0.16) × 10^5^	(1.16 ± 0.25) × 10^5^	83.1	(1.52 ± 0.30) × 10^5^	(1.58 ± 0.09) × 10^5^	103.8
824ccpA	(4.33 ± 2.63) × 10^4^	0	0	(4.60 ± 0.56) × 10^5^	(3.55 ± 2.87) × 10^4^	7.71	(4.46 ± 0.75) × 10^5^	(4.48 ± 1.43) × 10^4^	10.04	(2.93 ± 0.96) × 10^5^	(6.73 ± 5.29) × 10^4^	22.99

**Figure 7:**
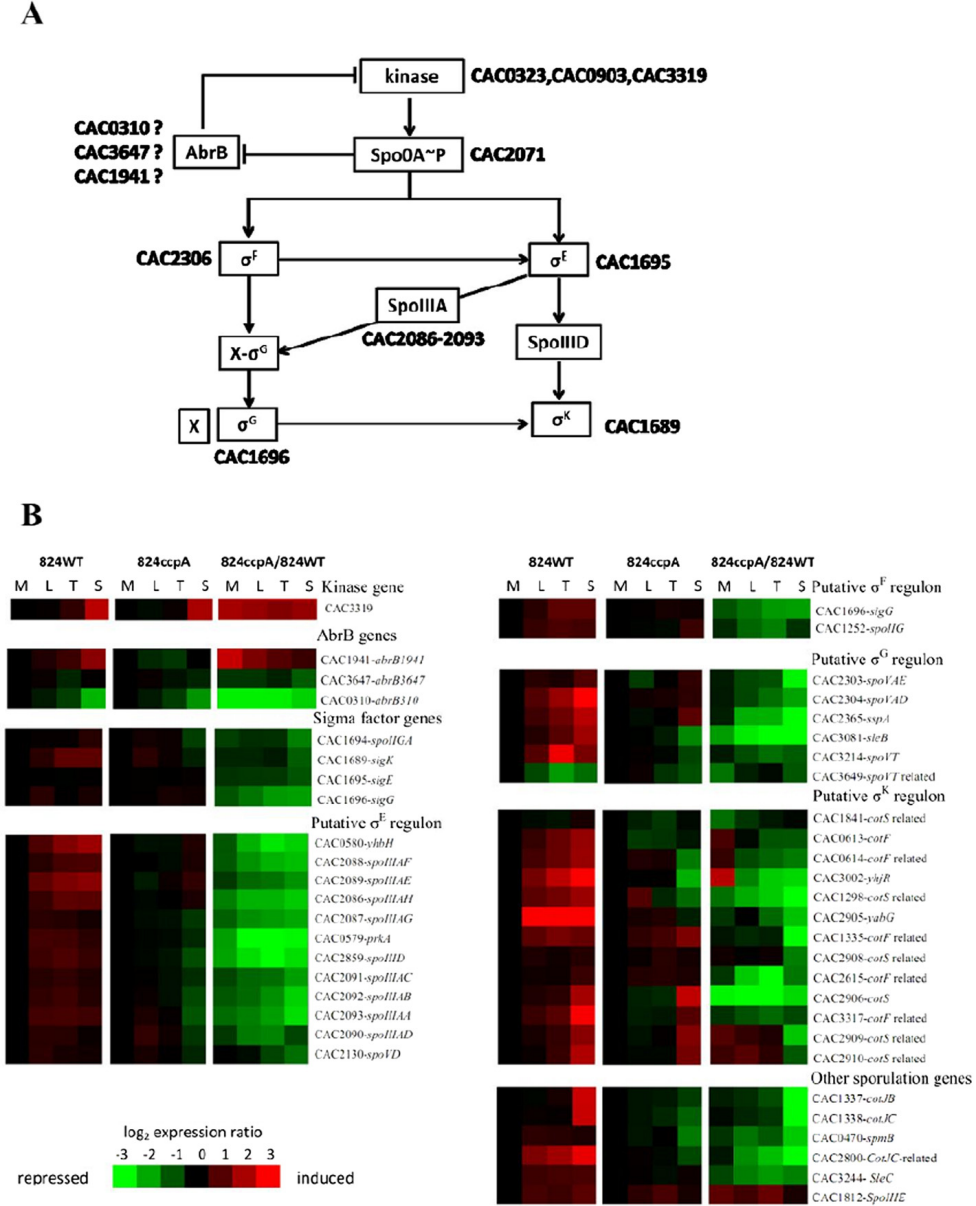
**Comparison of the transcriptional levels of sporulation-related genes between the 824ccpA and 824WT.** (**A**) the sporulation cascade proposed in *C. acetobutylicum*[77,78]*.* (**B**) Time-course study of expression profiles of the sporulation-related genes in the 824WT strain (left panel), the 824ccpA strain (middle panel) and between these two strains (right panel). Red and green indicate higher and lower expression, respectively. The reported values for time-course gene expression profile of 824WT strain or 824ccpA strain are the expression ratios compared to the first (middle-exponential) time point M.

It has been known that the phosphorylated form of Spo0A protein, namely Spo0A ~ P, is a key factor regulating sporulation process in *C. acetobutylicum*[71], and inactivation of *spo0A* blocked spore formation in *C. acetobutylicum*[71]. Three genes, encoding orphan histidine kinases (CAC0323, CAC0903 and CAC3319), were suggested to be responsible for transferring phosphoryl group to Spo0A [70]. However, microarray result here showed that, of these three genes, only CAC3319 exhibited increased expression level after *ccpA* inactivation (Table 2 and Figure 7B), which was contrary to the previous finding that inactivation of gene CAC3319 lowered sporulation to only 1% original level [70]. Therefore, whether these three orphan kinases played roles in CcpA-dependent regulation on sporulation still awaits further elucidation.

As mentioned above, three *abrB* genes (*abrB310*, *abrB1941* and *abrB3647*) exhibited transcriptional changes after *ccpA* inactivation. It has been reported that AbrB is a transcription factor involved in controlling the transition from vegetative growth to sporulation phase in *B. subtilis*[79,80]. Inactivation of the *abrB* gene significantly decreased sporulation in *B. subtilis*[80]. Similarly, it was found that inhibition of the *abrB310* translation by using antisense RNA techniques resulted in a delayed initiation of sporulation as well as the declined spore formation in *C. acetobutylicum*[63]. In addition, *abrB310* and *abrB3647* contain Spo0A-binding box in their promoter regions [68]. Thus, it is speculative that down-regulation of *abrB310* and *abrB3647* expression here may be one of the main reasons for deficient sporulation in *C. acetobutylicum ccpA* mutant.

The microarray result also showed that *ccpA* inactivation caused down-regulation of a set of genes encoding sporulation-specific sigma factors in *C. acetobutylicum* (Figure 7B), including *sigE* (CAC1695), *sigG* (CAC1696), *sigK* (CAC1689) and sigma factor E processing enzyme *SpoIIGA* (CAC1694) (Figure 7A). This suggested that CcpA may be one of the higher level regulators for these sporulation-related sigma factors. Early studies found that *sigE* and *sigF* inactivation caused sporulation stalling in *C. acetobutylicum*[81,82]. To further identify sporulation-related genes controlled by these sigma factors and CcpA in *C. acetobutylicum*, the well-established *B. subtilis* transcriptional regulation database, namely DBTBS ( http://dbtbs.hgc.jp/) [83], was used here. Most of the yielding genes exhibited down-regulation in the 824ccpA compare to the 824WT (Figure 7B), including: 11 σ^E^-controlled genes *prkA* (CAC0579), *yhbH* (CAC0580), *spoIIIAA-AH* (CAC2093-CAC2086), *spoVD* (CAC2130); 2 σ^F^-controlled genes *sigG* (CAC1696) and *spoIIG* (CAC1252); 13 σ^K^-controlled genes *cotF* (CAC0613), *cotF-*related genes (CAC0614, CAC1335, CAC2615, CAC3317), *cotS* (CAC2906), *cotS*-related genes (CAC1289, CAC1841, CAC2908-2910), *yabG* (CAC2905) and *yhjR* (CAC3002); 6 σ^G^-controlled genes *spoVAE* (CAC2303), *spoVAD* (CAC2304), *sspA* (CAC2365), *sleB* (CAC3081), *spoVT* (CAC3214) and *spoVT* homologue (CAC3649). Among these genes, only a putative σ^G^-controlled gene CAC3649 possesses an identifiable CRE site (Additional file 3), indicating that the majority of these sporulation-related genes may be indirectly regulated by CcpA. The result here also suggested that the σ-mediated spore formation processes in *C. acetobutylicum* may be similar with that in *B. subtilis*[84-89], and CcpA, as a key regulator, exerts a direct or cascade regulation on these genes.

## Conclusion

*C*. *acetobutylicum* has been used for industrial-scale butanol production for many decades. Although most of the relevant metabolic pathways have been deciphered, the complicated regulatory system in this strain remains unclear. In this study, we revealed the pleiotropic roles of a catabolite control protein CcpA in regulating several important physiological and biochemical processes in *C. acetobutylicum*, some of which are closely related to its industrial application. Inactivation of *ccpA* led to global expression changes of the genes for carbohydrate uptake and metabolism. Among the effects is the elimination of glucose repression on those non-preferable sugars. Our results showed that *C. acetobutylicum* CcpA may mediate the glucose repression by directly repressing genes for metabolizing these non-preferable carbon sources such as d-xylose and l-arabinose. This study has also showed that CcpA is involved in regulation of acidogenesis and solventogenesis and the transition of these two phases. The transcription profile analysis revealed that the normal expression of three important genes and gene clusters (i.e., *sol* operon, *bukII* and *abrB,*) related to acid and solvent production as well as transition, were all affected by *ccpA* inactivation. In addition, the impact of CcpA to efficient sporulation also suggested that CcpA might be a relative upper-level regulator compared to those known sporulation-related factors (e.g., sigma factors and *abrB* genes) in *C. acetobutylicum*. These findings not only improved our understanding of regulatory roles CcpA plays in solventogenic clostridia, but also provided some useful information for further genetic improvement of *C. acetobutylicum*.

## Methods

### Bacterial strains and growth conditions

The 824ccpA strain [11] was previously obtained by inactivating *ccpA* gene using TargeTron™ Gene Knockout System (Sigma-Aldrich, St. Louis, MO). *E. coli* was grown aerobically in LB medium at 37°C. 25 μg/mL chloramphenicol and 50 μg/mL kanamycin were used in LB medium when needed. The CGM medium [90] and P2 medium [91] were used for seed-growth and fermentation of *C. acetobutylicum*, respectively, under anaerobic condition at 37°C.

The P2 medium was used to cultivate *C. acetobutylicum* cells for microarray analysis. The CBM medium [92] was used for sporulation analysis. SMP2 medium, a synthetic minimal medium modified from P2 medium using glycerol and sodium pyruvate as the basic carbon sources, was adopted to grow *C. acetobutylicum* cells for the analysis of the “glucose effect” for *bukII* gene. The SMP2 components are listed as following: 20 g/L glycerol, 20 g/L sodium pyruvate, 6 g/L yeast extract, 0.5 g/L K_2_HPO_4_·H_2_O, 0.5 g/L KH_2_PO_4_, 2.2 g/L CH_3_COONH_4_, 0.2 g/L MgSO4·7H_2_O, 0.01 g/L MnSO_4_·H_2_O, 0.01 g/L NaCl, 0.01 g/L FeSO_4_·7H_2_O, 1 mg/L *p*-aminobenzoic acid, 1 mg/L thiamine, 0.01 mg/L biotin. d-glucose was supplied at a concentration of 20 g/L when investigating the “glucose effect” and all cells were collected at late exponential phase (A_600_ ~2.0).

### Fermentation conditions

The preinoculum and inoculum preparation were performed as described before [11]. For microarray and real-time reverse-transcript (RT)-PCR validation, the 824WT and 824ccpA were grown anaerobically in P2 medium at 37°C. The fermentations were conducted in BioFlo 110 bioreactors (New Brunswick Scientific, Edison, NJ) with 1.5 L working volumes, in which pH value was controlled over 5.0 by adding 9% (w/v) aqueous ammonia and mixed sugars (40g/L d-glucose and 20g/L d-xylose) were used as the carbon sources. In the fermentations without pH control, lower sugar concentration was adopted, and the fermentation was performed in 100 mL serum bottle with 50 mL working volume.

### Microarray analysis

Total RNA was extracted from the 824WT and 824ccpA strain, which were grown in the pH-controlled P2 medium containing 40 g/L d-glucose and 20 g/L d-xylose as the carbon sources, at four time points: middle exponential (time point M), late exponential (time point L), transition (time point T) and stationary phases (time point S) (Figure 1A). For investigating the glucose repression in the 824WT, samples were collected at time point S (d-glucose remained then) and S2 and S3 (d-glucose was exhausted at these two time points) (Additional file 1A). After centrifugation at 4°C, 5000 × *g* for 10 min, the cell pellets were collected and frozen immediately using liquid nitrogen and then ground into powder. Total RNA was extracted using Trizol™ (Invitrogen, Carlsbad, CA) and purified using RNeasy™ cleanup kit (Qiagen, Inc., Valencia, CA) according to the manufacturer’s instructions.

For microarray analysis, Algilent custom 60-mer oligonucleotide microarrays were used, in which a 60-mer oligonucleotide probe was used for each gene. Single-color microarray assays were performed by Shanghai Biochip Co. Ltd. (Shanghai, China) according to standard protocols provided by Agilent Technologies. In brief, RNA was reversely transcribed to cDNA using Moloney murine leukemia virus (MMLV) reverse transcriptase (Invitrogen, Carlsbad, CA). cDNA was transcribed with T7 RNA polymerase (New England BioLabs, Beverly, MA), resulting in aminoacyl-UTP (aaUTP, Ambion, Austin, TX)-labeled cRNA. cRNA was further labeled with Cy3 NHS ester (GE healthcare, Waukesha, WI) for both the 824WT and 824ccpA, and then hybridized to the probes (size: 60mer; three technical replicates for each ORF) on 8 × 15K custom 60-mer oligonucleotide chip as described previously [93]. Arrays were scanned with 5-μm resolution using an Agilent DNA Microarray scanner (G2565BA, Agilent Technologies, CA). The raw data was normalized across all arrays by quantile normalization [94] function from Feature Extraction software (Agilent Technologies, Santa Clara, CA) and then analyzed in GeneSpring GX (Agilent Technologies, Santa Clara, CA). To investigate expression fold changes of genes in the 824ccpA versus the 824WT, change-fold of every gene was obtained by dividing the normalized signal intensities of the same gene at each time point. 2-fold change was chosen as a threshold for differential expression. Average linkage hierarchical clustering was performed using Cluster 3.0 [95], and the resulting gene clusters were visualized in Java TreeView [96,97].

### Identification of CcpA-binding sites in *C. acetobutylicum*

The regulatory Sequence Analysis Tools (RSAT) [98] was used to retrieve the upstream non-coding regions of the genes that exhibit significant (≥2 fold) up-regulation and down-regulation after *ccpA* inactivation. Gibbs Motif Sampler program [30] was then used to discover the putative CcpA-binding sites, namely CRE (Catabolite Responsive Element) sites, located in these upstream regions based on a 16 bp “motif width” from the previous findings in *B. subtilis*[99,100] and *C. acetobutylicum*[57]. For those up-regulated genes, total 42 putative CRE sites were discovered through a “recursive” sampler mode of this method, thereby yielding a motif probability model, whereas no putative CRE sites were found for the down-regulated genes using this method. This motif probability model was then submitted to TOMTOM [101] for comparison against known motifs database RegTransBase [102] to see whether the newly discovered, putative CRE sites resembled previous discovered *B. subtilis* CRE motifs. A profile Hidden Markov Model (HMM) was constructed based on the training set (42 CRE sites obtained from Gibbs Motif Sampler analysis) with HmmBuild program included in HMMER 2.3 ( http://hmmer.janelia.org/) [31,103]. The profile HMM was then calibrated with HmmCalibrate program included in HMMER 2.3, resulting in a calibrated profile HMM. Then we used this calibrated profile HMM as a query to search the whole genome of *C. acetobutylicum* with HmmSearch program included in HMMER 2.3. The lowest positive score (i.e. =1.6), obtained from HMM search against the training set, was defined as the significance score. The scores for CRE sites were automatically assigned by the HmmSearch program. Two criteria were adopted here to yield putative CRE sites: *i*) sites with score over the significance score; *ii*) sites with score slightly lower than the significance score, but their corresponding genes exhibiting significant changes after *ccpA* inactivation. We removed some CRE sites based on the following criteria: *i*) the site was located at the end of ORF when an other CRE site with higher score was found in the 5’ -region of the ORF or found in the promoter; *ii*) the site was located in ORF of a gene which was positively regulated by CcpA, because, for those genes directly activated by CcpA, their CRE sites was normally found in the upstream region of promoter, such as *pta*[104], *ack*[105,106] and *ilvB*[107] in *B. subtilis*. The CRE sites alignment was performed through the MUSCLE [108] and the result was visualized using Weblog 3.2 [109].

### Analytical methods

The cell density (A_600_), residual sugars and metabolites (acids and ABE solvents) were determined using the methods described previously [11].

### Quantitative real-time RT-PCR analysis

The samples for microarray validation were collected from two independent cultures of *C. acetobutylicum*. RNA was extracted by using the procedure described previously [11]. cDNAs were synthesized using RTase M-MLV (RNase H^**-**^) reverse transcriptase (Takara, Japan). The PCR reaction was carried out in a Rotor-Gene 2000 real-time thermal cycling system (Corbett Research, Australia), using the following condition: 95°C for 2 min, followed by 40 cycles of 95°C for 15 s, 55°C for 20 s, and 72°C for 20 s. The pullulanase gene (CAC2679) was used as an internal control according to the previous report [110], and the microarray result here also indicated that CAC2679 had a constant expression profile in both the 824WT and 824ccpA strains (Additional file 2). Two biological repeats were performed for each gene and the change-fold was the mean ± SD of the two independent biological replicates. The primers used for real-time PCR were listed in Additional file 9: Table S5.

### Overexpression and purification of His_6_-tagged CcpA protein in *E. coli*

The *ccpA* gene was amplified from the genomic DNA of *C. acetobutylicum* ATCC 824 using the primer pair CAC3037-for/CAC3037-rev (Additional file 9). The amplified DNA was digested with *BamHI*/*Xho*I and cloned into pET28a (Novagen, Madison, WI) plasmid, yielding pET28a-ccpA. The resulting plasmid was transformed into *E. coli* Rosetta (DE3) (Novagen, Madison, WI). Gene expression was induced at 30°C for 3 hours by adding 1.0 mmol/L Isopropyl-β-D-thio-galactoside (IPTG) when A_600_ reached to 0.8. Cells were harvested by centrifugation (10 min, 5000 × *g*, 4°C), washed with a solution of 20 mM Tris–HCl, pH 7.9, 500 mM KCl, 10% (*v*/*v*) glycerol and 10 mM imidazole, and then disrupted by French Press (Constant Systems Limited, UK). Cell debris and membrance fractions were separated from the soluble fraction by centrifugation (20 min, 20, 000 × *g*, 4°C). The soluble fraction was loaded onto a Ni Sepharose™ 6 fast flow agrose (GE Healthcare, Waukesha, WI) column for purification of His_6_-CcpA protein. Protein was eluted by using a buffer (pH 7.9) containing 20 mM Tris–HCl, 500 mM KCl, 10% (*v*/*v*) glycerol and 200 mM imidazole. The eluent fraction was then transferred to Amicon Ultra-15 Centrifugal Filter (Milipore, Billerica, MA) and eluted for three times by using a buffer (pH 7.9) containing 20 mM Tris–HCl, 500 mM KCl, 10% (*v*/*v*) glycerol. The purified protein was stored at −80°C.

### Electrophoretic mobility shift assay (EMSA)

A universal primer (5’-AGCCAGTGGCGATAAG-3’) was labeled at 5’-terminal with Cyanine 5 (Cy5) (Biosune BioTec Inc., Shanghai). Cy5-labeled DNA probes were generated by two steps PCR amplification: first, double-stranded DNA fragments were amplified from the genomic DNA of *C. acetobutylicum* ATCC 824 using specific primer pairs containing universal primer sequence in the 5’-terminal; second, Cy5-tag was added to the above DNA fragments by PCR reaction using the universal primer labeled with Cy5. The resulting Cy5-labeled probes were recovered by agarose gel electrophoresis. EMSA was performed using a constant amount (~ 0.04 *p*mol) of labeled DNA probe.

His_6_-CcpA protein was pre-incubated with 0.04 *p*mol Cy5-labeled probe in a buffer containing 20 mM Tris–HCl, pH 7.9, 5% glycerol, 40 ng/mL bovine serum albumin (BSA), 0.25 mM DTT, 10 mM MgCl_2_, 20 mM KCl, and 50 ng/μL fish sperm DNA. The reaction mixture was incubated at room temperature for 15 min and then loaded on a 6% polyacrylamide (acrylamide: bis-acrylamide = 80:1) gel (20 μL per lane). The gel was first pre-run in 0.5 × TBE buffer at 120 V for 30 min in an ice-bath, then 20 μL of reaction mixture was loaded and electrophoresis continued for another 90 min. The gel was visualized using Starion FLA-9000 Scanner (FujiFilm, Japan).

### Sporulation assay

Sporulation analysis was performed according to the previous method [111]. The strains were grown in liquid CGM medium for 20–24 h. The same amount (A_600_) of the 824WT and 824ccpA cultures were then taken and spread onto CBM plates. At four time points (2, 4, 6 and 8d) of cultivation, all colonies on each CBM plate were collected, resuspended in 5 mL liquid CGM medium and quantified based on A_600_. The cell suspension was then halved: one part was subject to heat shock at 80°C for 10 min to kill all vegetative cells while the other part was not. 100 μL suspension of each treatment with appropriate dilution was spread onto the CGM plate again, and the plates were incubated anaerobically at 37°C for 48 h. Colonies on each CGM plate were counted. The sporulation rate was calculated based on the following formula:

$$\left({\text{colonies}}_{\text{heatshock}}/{\text{A}}_{600\text{heatshock}}\right)/\left({\text{colonies}}_{\text{non}-\text{heatshock}}/{\text{A}}_{600\text{non}-\text{heatshock}}\right)\text{.}$$

### Microarray data set accession number

The raw DNA microarray data set analyzed in this paper has been submitted to the NCBI Gene Expression Omnibus (GEO) database under the accession number GSE33364.

## Misc

Cong Ren and Yang Gu contributed equally to this work.

## Competing interests

The authors declare that they have no competing interests.

## Authors’ contributions

CR, YG, WJ, SY and WZ conceived of the study. WJ supervised the research, and participated in writing the manuscript. CR, YG and WZ drafted the manuscript. CR and YG carried out the genetic manipulation, fermentation, sporulation test, microarray experiment and EMSA analysis. YW did protein purification and participated in EMSA analysis. CR and CY did the bioinformatics analysis. All authors read and approved the final manuscript.

## Supplementary Material

Additional file 1 **Figure S1.** Comparison of the expression levels of the CcpA-repressed genes involved in carbohydrates metabolism in *C. acetobutylicum* ATCC 824 (824WT) in the presence and absence of d-glucose. Most of the CcpA-repressed genes exhibited significant upregulation when d-glucose was depleted (S2 and S3) in wild-type strain 824WT. Click here for file

Additional file 2: **Table S1.** Microarray data. The file lists the complete set of data from microarray experiment and the genes exhibiting 2-fold changes in the *ccpA* mutant (824ccpA) compared to its parental strain (824WT) at each time point. Click here for file

Additional file 3 **Table S2.** Identified putative CcpA-binding sites in *Clostridium acetobutylicum.* The table lists all of predicted catabolite repression elements (CREs) in *Clostridium acetobutylicum*. HMM method was used for the prediction. The training set for HMM search was obtained from microarray expression data. The genes up-regulated or down-regulated in transcript levels were indicated. Click here for file

Additional file 4 **Table S3.** List of the up-regulated genes involved in carbohydrates metabolism after *ccpA* inactivation and their putative CcpA binding sites (CREs). Click here for file

Additional file 5 **Table S4.** List of the down-regulated genes involved in carbohydrates metabolism after *ccpA* inactivation and their putative CcpA binding sites (CREs). Click here for file

Additional file 6 **Figure S2.** Genomic organization of the genes involved in pentose utilization and the corresponding binding loci of CcpA. Genes from the same operon are marked in the same color. Click here for file

Additional file 7 **Figure S3.** Quantitative RT-PCR analysis of gene *araR*, gene *araD* and gene *ptk* to assess the impact of l-arabinose on transcription of the gene cluster *araR-araD-araA1-ptk.* The fold difference in gene expression is calculated as the gene expression level in the cultures (grown in d-glucose-l-arabinose mixture) divided by that of the wild-type strain grown in l-arabinose. Click here for file

Additional file 8 **Figure S4.** Quantitative RT-PCR analysis to assess the impact of d-glucose on the expression of *bukII* (CAC1660) and *bukI* (CAC3075) in *C. acetobutylicum* ATCC 824. Cells were harvested from SMP2 medium with 20 g/L d-glucose (M + G) or without d-glucose (M) at late exponential phase (A600 = 2.0). Click here for file

Additional file 9 **Table S5.** primers used in this study. The file lists all of the primers used in this study. Click here for file
