# Supplementary material for: Pleiotropic functions of catabolite control protein CcpA in Butanol-producing Clostridium acetobutylicum
Source: BMC Genomics. 2012 Jul 30;13:349. doi: 10.1186/1471-2164-13-349 (PMC3507653; doi:10.1186/1471-2164-13-349)

Additional file 1. Comparison of the expression levels of the CcpA-repressed genes involved in carbohydrates metabolism in *C. acetobutylicum* ATCC 824 (824WT) in the presence (time point S in figure A) and absence of D-glucose (S2 and S3 in figure A). (A) Growth and sugar consumption of 824WT in fermenting D-glucose and D-xylose. Triangle, growth (A600); diamond, D-glucose concentration; square, D-xylose concentration. (B) Comparison of the expression level of CcpA-repressed genes involved in carbohydrates metabolism in the presence and absence of D-glucose. Most of the CcpA-repressed genes exhibited significant upregulation when D-glucose was depleted (S2 and S3) except for CAC3673, CAC2955 and CAC2962.

A

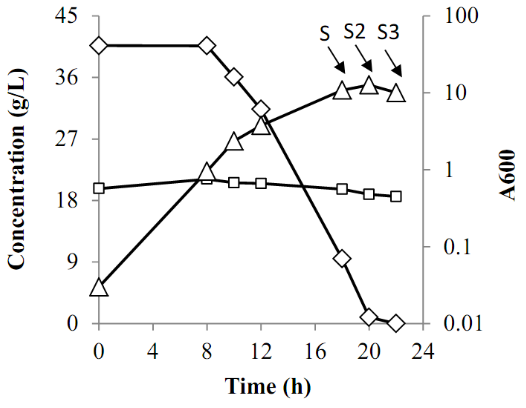

B

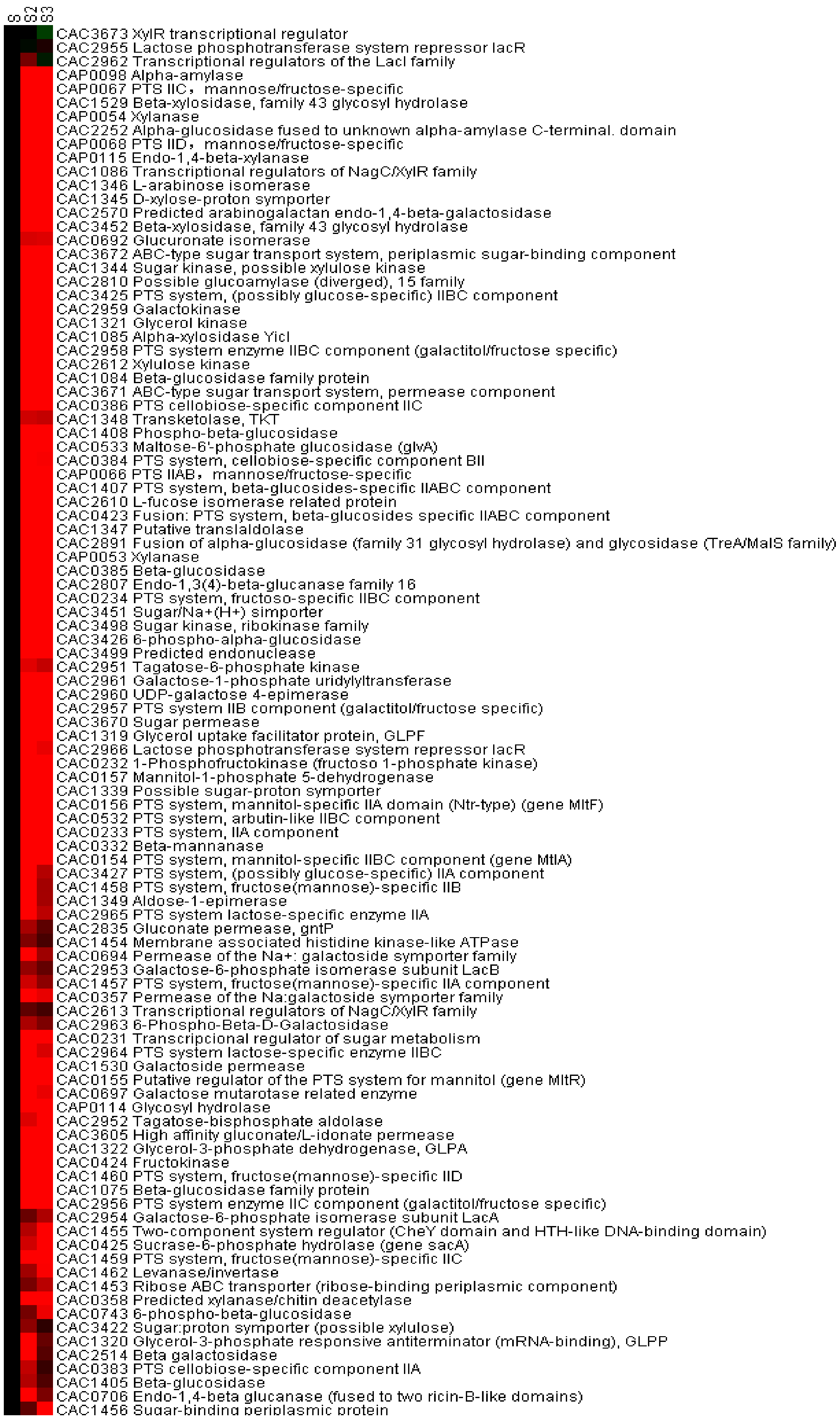

Supplement: Additional file 1 — Figure S1. Comparison of the expression levels of the CcpA-repressed genes involved in carbohydrates metabolism in C. acetobutylicum ATCC 824 (824WT) in the presence and absence of d-glucose. Most of the CcpA-repressed genes exhibited significant upregulation when d-glucose was depleted (S2 and S3) in wild-type strain 824WT. [file 1471-2164-13-349-S1.pdf]
