# Supplementary material for: Pleiotropic functions of catabolite control protein CcpA in Butanol-producing Clostridium acetobutylicum
Source: BMC Genomics. 2012 Jul 30;13:349. doi: 10.1186/1471-2164-13-349 (PMC3507653; doi:10.1186/1471-2164-13-349)
Supplement: Additional file 4 — Table S3. List of the up-regulated genes involved in carbohydrates metabolism after ccpA inactivation and their putative CcpA binding sites (CREs). [file 1471-2164-13-349-S4.pdf]

Additional file 4. List of the up-regulated genes involved in carbohydrates metabolism after *ccpA* inactivation as well as their putative CcpA binding sites (CREs)[§].

Gene locus	Annotation	Gene name	Expression ratio (824ccpA/824WT)				CcpA binding sites (CREs)		
			M	L	T	S	Sequence	Position[†]	Score
CAP0053	Xylanase	*xynB*		2.1		12.2			
CAP0054	Xylanase/chitin deacetylase family protein		3.1	6.5	2.4	12.9	ttgaatacctttcaa	-110	4.4
							atgtaaacacattcat	-56	3.2
CAP0066	PTS IIAB, mannose/fructose-specific	*ptnA*	32.8	60.0	4.5	27.5	atgaaaacgtattaat	-321	6.4
CAP0067	PTS IIC, mannose/fructose-specific		31.8	64.3	4.7	30.0			
CAP0068	PTS IID, mannose/fructose-specific		35.6	55.4	3.9	26.8			
CAP0098	Alpha-amylase	*amyA1*	235.5	410.7	40.9	783.2	aagaaaaagcttactt	21	2.0
							aagtaatcgttttcaa	87	7.0
CAP0114	Glycosyl hydrolase		6.2	5.5	2.2	6.6			
CAP0115	Endo-1,4-beta-xylanase	*xynD*	6.0	4.7	2.0	5.7	gtgtaatcgattttaa	800	3.1
CAC0154	PTS system, mannitol-specific IIBC	*MtlA*	24.9	18.2	2.7	16.9	ttgaaaacgtatacca	-112	3.8
							ttgaaaacgatcgcat	-78	4.5
CAC0155	Regulator of the PTS system for mannitol	*MtlR*	11.6	20.3	2.4	11.2	atttaaatgattacaa	863	1.9
CAC0156	PTS system, mannitol-specific IIA	*MltF*	7.4	9.5	2.0	9.1			
CAC0157	Mannitol-1-phosphate 5-dehydrogenase	*MtlD*	9.3	9.4		6.9	ttgaaaatgaatactt	839	2.2
CAC0231	Transcripcional regulator of sugar metabolism		5.9	14.0	4.8	16.2	ttgaatccgtttacaa	-79	3.5
CAC0232	1-Phosphofructokinase	*fruB*	10.6	18.4	4.3	21.4	ttgtagacgatttaaa	278	1.5
CAC0233	PTS system IIA, fructoso-specific		15.2		2.5	25.8	aagaaaacttatgcat	363	1.6
CAC0234	PTS system IIBC, fructoso-specific		7.1	9.7		17.6			
CAC0332	Beta-mannanase	*manB*	5.7	2.9	5.2	48.1	ttgtaaacgtttttat	-158	8.5
CAC0357	Permease of the Na:galactoside symporter		4.0	2.8	2.7	3.2			
CAC0358	Xylanase/chitin deacetylase		3.4	4.2	4.4	5.8			

CAC0383	PTS IIA, cellobiose-specific		6.0	12.0					
CAC0384	PTS IIB, cellobiose-specific	*licB*	5.8	13.9	0.5				
CAC0385	Beta-glucosidase		4.1	10.2					
CAC0386	PTS IIC, cellobiose-specific	*licC*	3.7	9.4	0.3				
CAC0423	PTS IIBCA, beta-glucosides specific	*scrA*	7.1	5.3		2.6			
CAC0424	Fructokinase	*scrK*	7.4	5.0		3.1	ttttaatcgattttat	29	2.6
CAC0425	Sucrase-6-phosphate hydrolase	*sacA*	2.8	2.2					
CAC0532	PTS system IICB, maltose specific		19.6	16.1	16.2	19.1	ttgaaaaggttttctt	-202	5.6
CAC0533	Maltose-6'-phosphate glucosidase	*glvA*	16.6	16.8	6.5	14.4			
CAC0569	Antiterminator	*glcT*	3.2	2.7	2.5	3.6			
CAC0692	Glucuronate isomerase	*uxaC*	3.7	3.1		2.7	gtgataacgtttactc	-361	1.9
CAC0694	Na: galactoside symporter		4.3	4.1	3.4	4.1			
CAC0697	Galactose mutarotase related enzyme		6.8	6.2	3.0	2.7			
CAC0706	Endo-1,4-beta glucanase		6.1	7.4	5.5	8.0	cagataacgtttacaa	-76	3.4
CAC0743	6-Phospho-beta-glucosidase		3.5	3.2	3.2	3.0	atgataaccttatcat	-151	4.2
CAC1075	Beta-glucosidase		5.4	7.0	4.8	47.7	ttgtaaatgttttcat	-50	7.3
CAC1084	Beta-glucosidase		14.0	16.1	12.1	12.6	atgtatacgatttctt	-102	6.0
CAC1085	Alpha-xylosidase		13.3	14.0	7.0	13.0			
CAC1086	NagC/XylR family transcriptional regulator	*xlyR*	9.2	10.8	4.9	12.0			
CAC1319	Glycerol uptake facilitator protein	*glpF*	32.7	32.1	52.9	60.0	gagtaaacatttacat	-311	3.7
CAC1320	Glycerol-3-phosphate responsive antiterminator	*glpP*	11.3	14.2	11.9	7.9			
CAC1321	Glycerol kinase	*glpK*	35.0	30.4	39.3	47.3	atgtaaccgattacac	536	2.9
CAC1322	Glycerol-3-phosphate dehydrogenase	*glpA*	2.5	2.9	2.1	2.1			
CAC1339	Sugar-proton symporter	*araE*	35.3	43.3	11.6	36.2	aggtaatcgctttcat	-150	4.4
							atgaaaacgttatata	-91	4.7

CAC1341	L-ribulose-5-phosphate 4-epimerase	*araD*	2.3			5.8	tggtaaacgttttact	-105	1.5
CAC1342	L-arabinose isomerase	*araA1*	2.4			2.6			
CAC1343	Phosphoketolase	*ptk*				2.9			
CAC1344	Ribulokinase	*xylB*	13.0	12.2	17.5	49.7	ttgcaaacgatttctc	95	2.7
CAC1345	D-Xylose-proton symporter	*xylT*	71.6	70.2	33.6	147.4	atgaaatcgtttaaaa	-61	5.5
CAC1346	L-Arabinose isomerase	*araA*	41.4	39.2	20.1	118.1			
CAC1347	Translaldolase	*tal*	6.6	4.7	3.8	6.9			
CAC1348	Transketolase	*tkt*	5.6	3.7	3.9	7.9			
CAC1349	Aldose-1-epimerase	*galM*	5.3	4.2	3.1	7.2			
CAC1405	Beta-glucosidase	*bglA*	5.8	2.8		3.5	atgataacgtattcga	326	3.0
CAC1406	Transcriptional antiterminator (BglG family)		26.1	28.8	12.0	40.2			
CAC1407	PTS IIBCA, beta-glucosides-specific		26.6	24.4	12.6	30.9	aagaaaacgttaacag	50	4.7
							ttgtaaccgttatcaa	152	4.6
CAC1408	Phospho-beta-glucosidase		2.9	2.1	2.3	2.1	gagaaaactatttctt	782	2.4
CAC1453	Periplasmic fructose-binding protein*		3.4	4.1	3.7	3.7			
CAC1454	Histidine kinase		3.3	4.4	3.7	4.8	ttgtaaaccttatcat	47	5.9
CAC1455	Two-component system regulator								
CAC1456	periplasmic fructose-binding protein*		2.1	2.8	2.1	2.6			
CAC1457	PTS IIA, fructose/mannose specific		14.0	6.5	5.7	16.9	ttgaaaacgttacaa	158	3.7
CAC1458	PTS IIB, fructose/mannose specific		14.7	6.3	5.6	40.9			
CAC1459	PTS IIC, fructose/mannose specific		7.6	4.1	3.2	7.6	atgaaaactttattta	583	1.0
CAC1460	PTS IID, fructose/mannose specific		7.1	3.4	2.6	7.2			
CAC1462	Levanase/invertase		5.6	5.3	6.0	4.2	ttgaaaacgtttttat	-51	9.1
CAC1529	Alpha-L-arabinofuranosidase II precursor *	*arb43*	7.6	10.0	11.1	15.2			
CAC1530	Sugar-proton symporter, possibly for arabinosides [1]	*araT*	10.9	15.3	14.6	22.0	ttgtaagcgttatcac	1169	2.3
CAC2252	Alpha-glucosidase		17.0	28.4	11.9	296.2	gtgaaatcgattgcaa	-84	3.7

							atgaaaacgatatcta	121	6.8
CAC2018	Aldehyde:ferredoxin oxidoreductase		4.2	4.4	3.2	2.7			
CAC2514	Beta galactosidase		38.9	46.3	15.0	69.9	attaaaaggaattcat	156	1.0
							atgaaaacgttttata	947	6.3
CAC2570	Arabinogalactan endo-1,4-beta-galactosidase	*ganA*	5.8	6.8	3.3	11.0	tagaaaacgatttctt	-56	7.9
CAC2610	Xylose isomerase	*xlyA*	35.6	49.0	6.6	52.4			
CAC2611	Hypothetical protein		35.2	40.5	7.4	27.2			
CAC2612	Xylulose kinase	*xylB*	26.9	30.3	6.1	17.9	atgaaaaagtttacaa	-104	5.9
CAC2613	Glucokinase*	*glcK*	2.6	2.7	2.3	2.8	attaaaacggttactt	634	1.8
CAC2807	Endo-1,3(4)-beta-glucanase		5.7	11.2	4.1	11.2			
CAC2810	Glucoamylase		11.7	23.7	7.7	98.7	atgataacggttacaa	1340	3.4
CAC2835	Galactose permease	*gntP*	93.9	143.6	49.3	321.9	aagaaaacgttaccgt	-32	2.4
CAC2891	Alpha-glucosidase		3.5	6.9	2.8	9.8	gtgaaaacgtttaata	-123	3.8
							gggaaaacgattgcat	-73	3.5
CAC2951	Tagatose-6-phosphate kinase	*lacC*		2.1					
CAC2952	Tagatose-bisphosphate aldolase	*gatY*		2.1					
CAC2953	Galactose-6-phosphate isomerase subunit B	*lacB*		2.6	2.0	2.2			
CAC2954	Galactose-6-phosphate isomerase subunit A	*lacA*		2.5	2.1	2.4			
CAC2955	Lactose phosphotransferase system repressor	*lacR*		2.3	3.5	3.7			
CAC2956	PTS IIC, galactose specific		6.8	7.3	7.1	11.4	atgtatacgttatttt	298	2.4
							ttgtaaatgttataat	332	2.7
CAC2957	PTS IIB, galactose specific		7.9	17.6	8.6	25.1			
CAC2958	PTS IIA, galactose specific		4.6	5.7	4.7	9.1	tagtaatcgcttacgt	-315	2.6
							atgtaaacgaaatcat	-89	6.3

CAC2959	Galactokinase	*galK*	13.2	9.7	3.0	9.3	atgatttcgtttacat	-387	4.1
							tagtaatcgattacta	-40	3.8
							atgaaaatgttttctt	56	6.2
CAC2960	UDP-galactose 4-epimerase	*galE*	5.8	5.3	3.1	8.2			
CAC2961	Galactose-1-phosphate uridylyltransferase	*galT*	2.0	2.0	2.0	4.6			
CAC2962	Transcriptional regulator					3.0			
CAC2963	6-Phospho-Beta-D-Galactosidase	*lacG*	2.4	2.4	2.2	2.1			
CAC2964	PTS system lactose-specific enzyme IIBC	*lacE*	4.7	3.8	2.9	3.3			
CAC2965	PTS system lactose-specific enzyme IIA	*lacF*	12.6	6.7	6.2	10.0			
CAC2966	Lactose phosphotransferase system repressor	*lacR*		2.0	2.8		atgtaaacgaaaacaa	-134	4.8
							tagaaaacgataacta	-77	4.8
CAC3422	Sugar:proton symporter ,possible xylulose		8.4	9.1	4.7	4.5			
CAC3425	PTS system, IIBC component	*glvC*	25.0	25.0	7.0	25.9	aagtaaacattttcac	-92	3.1
							cattaatcgtttacat	260	1.0
CAC3426	6-Phospho-alpha-glucosidase	*glvG*	5.4	4.7	4.2	11.0	atgaaaaagtattcaa	1	3.8
CAC3427	PTS system, IIA component		2.1	2.0	2.3	5.0	aggaaaacttttaaat	63	1.7
CAC3451	Sugar/Na(H) simporter	*XynT*	52.9	113.7	14.9	199.7	ttgaaaaagtttgtat	-69	2.2
							atgtaaataattacaa	-35	1.4
CAC3452	Beta-xylosidase	*xynB [2]*	43.2	100.5	14.1	189.5			
CAC3498	Fructoselysine kinase*		27.4	19.7	5.9	46.6	atgaaaacgattttga	329	4.8
CAC3499	Endonuclease		26.3	22.1	8.2	47.4			
CAC3500	anaerobic C4-dicarboxylate transporter	*dcuB*	13.61	13.61	8.50	19.33	ttgaaaacgattgtat	-261	5.9
CAC3605	High affinity glucxonate/L-idonate permease	*gntP*	10.3	4.8	2.2	3.9	tttaaaacgttttgat	588	3.4
								62	2.7

CAC3670 ↑	Sugar permease	*amyC*	95.3	94.1	33.6	141.2			
CAC3671	ABC transporter, permease component	*amyD*	158.3	163.4	46.5	295.8	atgataacgatttctc	206	3.8
CAC3672	ABC transporter, periplasmic sugar-binding component	*msmE*	98.7	71.2	41.7	126.5	atgtaaacgatttaat	-45	7.8

Notes:

§ The arrows indicate the transcription direction of the predicted operon obtained from DOOR database [3, 4], except for CAC1344-CAC1349 and CAC2610-CAC2612, which were identified to be one transcriptional unit by Zhang *et al.* [1] and this study (data not shown), respectively.

† The +1 position is defined as the first nucleotide of coding sequence.

* Annotated in SEED annotation system [5].

References

1. Zhang L, Leyn SA, Gu Y, Jiang W, Rodionov DA, Yang C: **Ribulokinase and transcriptional regulation of arabinose metabolism in Clostridium acetobutylicum**. *Journal of bacteriology* 2012, **194**(5):1055-1064.
2. Gu Y, Ding Y, Ren C, Sun Z, Rodionov DA, Zhang W, Yang S, Yang C, Jiang W: **Reconstruction of xylose utilization pathway and regulons in Firmicutes**. *BMC Genomics* 2010, **11**:255.
3. Mao F, Dam P, Chou J, Olman V, Xu Y: **DOOR: a database for prokaryotic operons**. *Nucleic Acids Res* 2009, **37**(Database issue):D459-463.
4. Dam P, Olman V, Harris K, Su ZC, Xu Y: **Operon prediction using both genome-specific and general genomic information**. *Nucleic Acids Research* 2007, **35**(1):288-298.
5. Overbeek R, Begley T, Butler RM, Choudhuri JV, Chuang HY, Cohoon M, de Crecy-Lagard V, Diaz N, Disz T, Edwards R *et al*: **The subsystems approach to genome annotation and its use in the project to annotate 1000 genomes**. *Nucleic Acids Res* 2005, **33**(17):5691-5702.
