# Supplementary material for: Pleiotropic functions of catabolite control protein CcpA in Butanol-producing Clostridium acetobutylicum
Source: BMC Genomics. 2012 Jul 30;13:349. doi: 10.1186/1471-2164-13-349 (PMC3507653; doi:10.1186/1471-2164-13-349)
Supplement: Additional file 5 — Table S4. List of the down-regulated genes involved in carbohydrates metabolism after ccpA inactivation and their putative CcpA binding sites (CREs). [file 1471-2164-13-349-S5.pdf]

Additional file 5. List of the down-regulated genes involved in carbohydrates metabolism after *ccpA* inactivation and their putative CcpA binding sites (CREs) [§]

Gene locus	Annotation	Gene name	Expression ratio (824ccpA/824WT)				*CcpA*-binding sites (CREs)		
			M	L	T	S	Sequence	Position[†]	Score
CAC0539	Mannanase-like protein, contains ChW-repeats	*manB*	0.11	0.14	0.22	0.17			
CAC0540	Mannanase-like protein, contains ChW-repeats		0.10	0.13	0.26	0.22			
CAC0570	PTS IIABC, glucose specific	*glcG*	0.16	0.20	0.11	0.19			
CAC1351	Periplasmic sugar-binding protein		0.21	0.30	0.22				
CAC1353	PTS IICB, glucose specific		0.07	0.07	0.11	0.20	ttgtaaacgatgtaaa	-219	1.7
CAC1354	PTS IIA, glucose specific		0.25	0.44			tttacatcgtttacaa	-214	1.7
CAC2237	Glucose-1-phosphate adenylyltransferase	*glgC*	0.44	0.37	0.43	0.34			
CAC2238	ADP-glucose pyrophosphorylase	*glgC*	0.51	0.35	0.41	0.33			
CAC2239	Glycogen synthase	*glgA*		0.39	0.47	0.48			
CAC2240	Cyclomaltodextrin glucanotransferase			0.44	0.39	0.48			
CAC3087	Phosphoenolpyruvate-protein kinase	*ptsI*	0.32		0.11	0.17			

Notes:

[§] The black arrows indicate the transcription direction of the predicted operon obtained from DOOR database [1, 2].

[†] The +1 position is defined as the first nucleotide of coding sequence.

References

1. Mao F, Dam P, Chou J, Olman V, Xu Y: **DOOR: a database for prokaryotic operons.** *Nucleic Acids Res* 2009, **37:**D459-463.
2. Dam P, Olman V, Harris K, Su ZC, Xu Y: **Operon prediction using both genome-specific and general genomic information.** *Nucleic Acids Research* 2007, **35:**288-298.
