# Supplementary material for: Pleiotropic functions of catabolite control protein CcpA in Butanol-producing Clostridium acetobutylicum
Source: BMC Genomics. 2012 Jul 30;13:349. doi: 10.1186/1471-2164-13-349 (PMC3507653; doi:10.1186/1471-2164-13-349)
Supplement: Additional file 6 — Figure S2. Genomic organization of the genes involved in pentose utilization and the corresponding binding loci of CcpA. Genes from the same operon are marked in the same color. [file 1471-2164-13-349-S6.pdf]

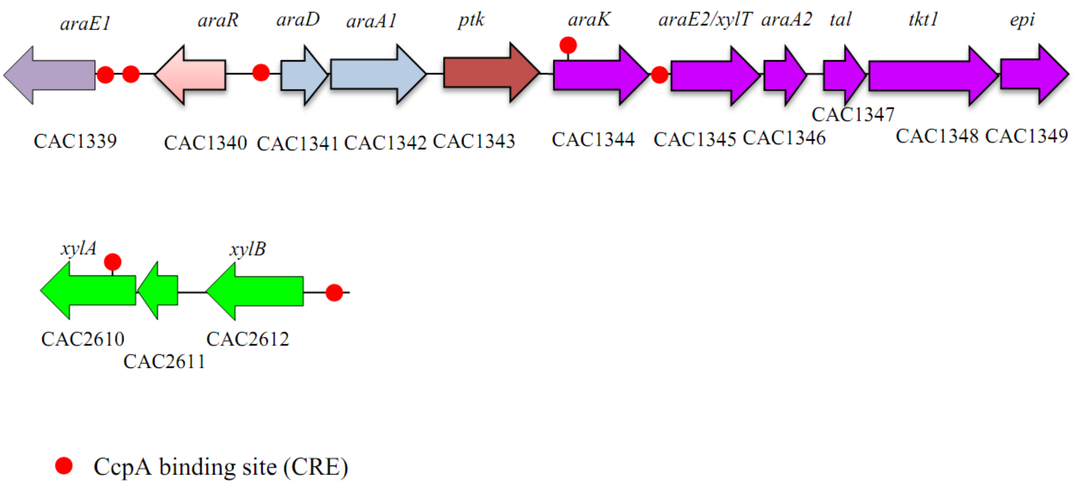

Additional file 6. Genomic organization of genes involved in pentose utilization and the corresponding binding loci of CcpA. Genes (shown by arrows) from the same operon are marked in the same colors. The binding sites of CcpA are shown by red circles.
