# Supplementary material for: Pleiotropic functions of catabolite control protein CcpA in Butanol-producing Clostridium acetobutylicum
Source: BMC Genomics. 2012 Jul 30;13:349. doi: 10.1186/1471-2164-13-349 (PMC3507653; doi:10.1186/1471-2164-13-349)
Supplement: Additional file 7 — Figure S3. Quantitative RT-PCR analysis of gene araR, gene araD and gene ptk to assess the impact of l-arabinose on transcription of the gene cluster araR-araD-araA1-ptk. The fold difference in gene expression is calculated as the gene expression level in the cultures (grown in d-glucose-l-arabinose mixture) divided by that of the wild-type strain grown in l-arabinose. [file 1471-2164-13-349-S7.pdf]

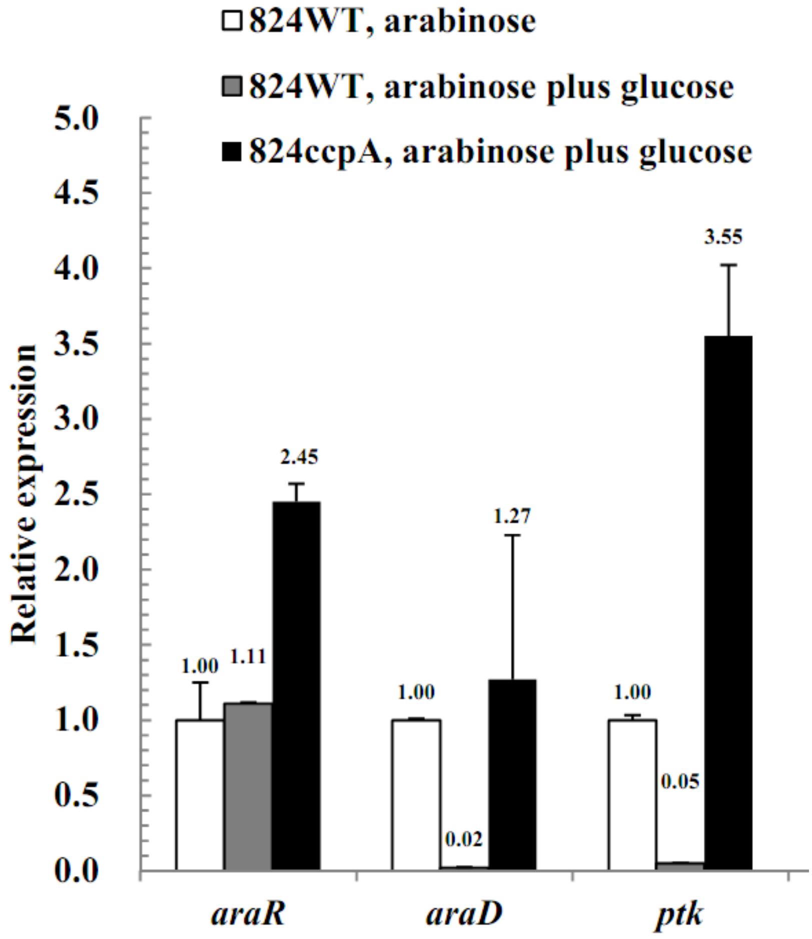

Additional file 7. Quantitative RT-PCR analysis of gene *araR*, *araD* and gene *ptk* to assess the effect of L-arabinose on the transcription of gene cluster *araR-araD-araA1-ptk*. Total RNA was isolated from *C. acetobutylicum* ATCC 824 wild-type (824WT) and its *ccpA*-inactivated strain (824ccpA). The cells were harvested from P2 medium [without pH controlling and using L-arabinose or L-arabinose-D-glucose mixture (D-glucose, 20 g/L; L-arabinose, 10 g/L) as the carbon sources] at the middle exponential phase (A_{600} ≈1.5). The expression levels of each gene were normalized to the gene expression in the wild-type strain grown on L-arabinose.
