# Supplementary material for: Pleiotropic functions of catabolite control protein CcpA in Butanol-producing Clostridium acetobutylicum
Source: BMC Genomics. 2012 Jul 30;13:349. doi: 10.1186/1471-2164-13-349 (PMC3507653; doi:10.1186/1471-2164-13-349)
Supplement: Additional file 8 — Figure S4. Quantitative RT-PCR analysis to assess the impact of d-glucose on the expression of bukII (CAC1660) and bukI (CAC3075) in C. acetobutylicum ATCC 824. Cells were harvested from SMP2 medium with 20 g/L d-glucose (M + G) or without d-glucose (M) at late exponential phase (A600 = 2.0). [file 1471-2164-13-349-S8.pdf]

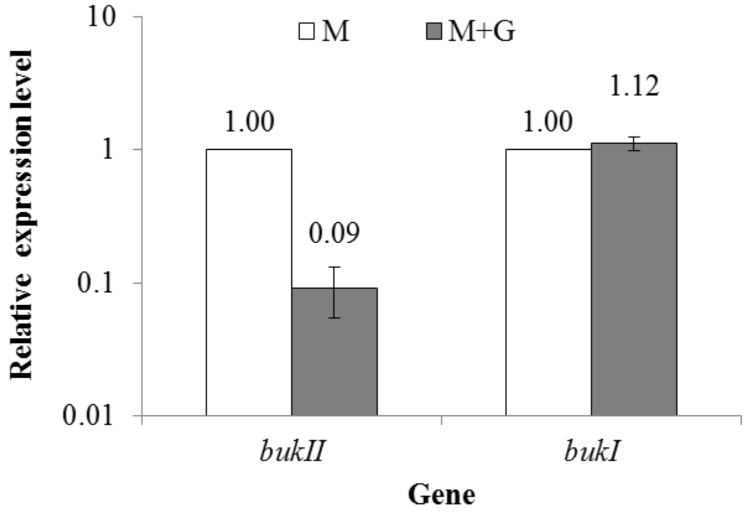

Additional file 8. Quantitative RT-PCR analysis to assess the impact of D-glucose on the expression of *bukII* (CAC1660) and *bukI* (CAC3075) in *C. acetobutylicum* ATCC 824. Cells were harvested from SMP2 medium with 20 g/L D-glucose (M+G) or without D-glucose (M) at late exponential phase (A_{600} = 2.0).
