# Supplementary material for: Pleiotropic functions of catabolite control protein CcpA in Butanol-producing Clostridium acetobutylicum
Source: BMC Genomics. 2012 Jul 30;13:349. doi: 10.1186/1471-2164-13-349 (PMC3507653; doi:10.1186/1471-2164-13-349)
Supplement: Additional file 9 — Table S5. primers used in this study. The file lists all of the primers used in this study. [file 1471-2164-13-349-S9.pdf]

Additional file 9. Primers used in this study.

Primer name	Sequence (5′-3′)	Description
Psol(EMSA)-for	AGCCAGTGGCGATAAGTAGGAAAACTGCTAAATG	forward EMSA probe primer for promoter of *sol* operon (CAP0162-64)
Psol(EMSA)-rev	AGCCAGTGGCGATAAGAAATATACACTTCTTTCTAAAAT	reverse EMSA probe primer for promoter of *sol* operon (CAP0162-64)
PxylB(EMSA)-for	AGCCAGTGGCGATAAGAAAGACTGCTCGAAAATT	forward EMSA probe primer for promoter of *xylB* (CAC2612)
PxylB(EMSA)-rev	AGCCAGTGGCGATAAGGCTGTCTTGGTTCCTGAT	reverse EMSA probe primer for promoter of *xylB* (CAC2612)
ParaE(EMSA)-for	AGCCAGTGGCGATAAGCACATAAATATTATGATTTAT	forward EMSA probe primer for promoter of *araE* (CAC1339)
ParaE(EMSA)-rev	AGCCAGTGGCGATAAGAACTCCTCCTTAAGATT	reverse EMSA probe primer for promoter of *araE* (CAC1339)
CAC1790-for	AGCCAGTGGCGATAAGGGGGTTGAATCCATCTA	forward primer for non-specific competitor in EMSA analysis
CAC1790-rev	AGCCAGTGGCGATAAGAAAGCTACCTTGCCATC	reverse primer for non-specific competitor in EMSA analysis
Cy5-tag primer	Cy5-AGCCAGTGGCGATAAG	universal primer for adding Cy5 tag
CAC3037-for	CGGGATCCATGGCTGCCTCTATTAAAG	forward primer for expression of CcpA in *E.coli*
CAC3037-rev	CCCTCGAGTTTTGCGTGAGGGTTTTAC	reverse primer for expression of CcpA in *E.coli*
qCAC1345-for	AGCTGTAGGTGGAGCTTCTGCTTT	forward qRT-PCR primer for *xylT* (CAC1345)
qCAC1345-rev	TCCAACGCCATCCTTCAAATGCAC	reverse qRT-PCR primer for *xylT*(CAC1345)
qCAC2612-for	AGTAAGTGGTGGAGGTG	forward qRT-PCR primer for *xlyB* (CAC2612)
qCAC2612-rev	ATTGCTACGCCAAGTGC	reverse qRT-PCR primer for *xylB* (CAC2612)
qCAC1348-for	AATGCTGTGGGAATGGCAATAGCG	forward qRT-PCR primer for *tkt* (CAC1348)
qCAC1348-rev	TGCAAGAGAACTTGCCTCTCCTGA	reverse qRT-PCR primer for *tkt* (CAC1348)
qCAC1941-fw	GGAATAGTAAGAAAATTGGATAG	forward qRT-PCR primer for *abrB1941* (CAC1941)
qCAC1941-rev	GCTTCACCGCAGAACAC	reverse qRT-PCR primer for abrB1941 (CAC1941)
qCAC1660-for	TGGAGGTATAGCCTACAATAAG	forward qRT-PCR primer for *bukII* (CAC1660)
qCAC1660-rev	CCTTCTGCTAGTGCTAACAT	reverse qRT-PCR primer for *bukII* (CAC1660)
qCAC3319-for	AGAAGCGGAGGTACAGG	forward qRT-PCR primer for orphan kinase CAC3319
qCAC3319-rev	TTGGATTTGCTATGGGT	reverse qRT-PCR primer for orphan kinase CAC3319

qCAC3673-for	CTGGAATAGTGATTGAGGGACAGC	forward qRT-PCR primer for *xylR* (CAC3673)
qCAC3673-rev	AGATGCATACATTTCCCAGCACCC	reverse qRT-PCR primer for *xylR* (CAC3673)
qCAC2966-for	TTTGAGGAGCTTTCACCAAGTG	forward qRT-PCR primer for *lacR* (CAC2966)
qCAC2966-rev	CCTTTATAAGACCTGCTGCTAGCCT	reverse qRT-PCR primer for *lacR* (CAC2966)
qCAC3647-fw	AGAACATTAGAAATAGCAGAG	forward qRT-PCR primer for *abrB3647* (CAC3647)
qCAC3647-rev	GCATCTCCACAGAAAATAC	reverse qRT-PCR primer for *abrB3647* (CAC3647)
qCAC1653-for	AGCATCCACCTGAAATG	forward qRT-PCR primer for glycosyltransferase CAC1653
qCAC1653-rev	TTACCTCCAAGCACAAA	reverse qRT-PCR primer for glycosyltransferase CAC1653
qCAP0164-for	ACGGAATAGTTGGAATGGGCGCTA	forward qRT-PCR primer for *sol* operon(CAP0162-64)
qCAP0164-rev	TGTGCCGTCAGGAAGTACTGTTGT	reverse qRT-PCR primer for *sol* operon(CAP0162-64)
qCAC0103-fw	TTTTAGGGAAAGATTTTGTAGAG	forward qRT-PCR primer for *cysC* (CAC0103)
qCAC0103-rev	TATGCCTTTTGGGTCTCG	reverse qRT-PCR primer for *cysC* (CAC0103)
qCAC0570-for	AAGGTGCTTGAAGCTATAGGCGGA	forward qRT-PCR primer for glucose-PTS system CAC0570
qCAC0570-rev	CTGTCATTACTCCAGCTGCTCCAA	reverse qRT-PCR primer for glucose-PTS system CAC0570
qCAC0310-fw	TTTATGTAGATGGTGAGCAA	forward qRT-PCR primer for *abrB310* (CAC0310)
qCAC0310-rev	ATAGTTGATAACATCGCTTG	reverse qRT-PCR primer for *abrB310* (CAC0310)
qCAC1340-fw	TGGAGGACACGCTTTATTC	forward qRT-PCR primer for *araR* (CAC1340)
qCAC1340-rev	TATGAGGCATTTAGGGTTAC	reverse qRT-PCR primer for *araR* (CAC1340)
qCAC1341-fw	TTCACCTTGGGCTACATC	forward qRT-PCR primer for *araD* (CAC1341)
qCAC1341-rev	CTGGAACATCATTTGGG	reverse qRT-PCR primer for *araD* (CAC1341)
qCAC1343-fw	CCAGCAGATGCCAATACC	forward qRT-PCR primer for *ptk* (CAC1343)
qCAC1343-rev	TTACTTGCCCAATCCCAAATG	reverse qRT-PCR primer for *ptk* (CAC1343)
